# The RhoA dependent anti-metastatic function of RKIP in breast cancer

**DOI:** 10.1038/s41598-021-96709-6

**Published:** 2021-08-31

**Authors:** Gardiyawasam Kalpana, Christopher Figy, Jingwei Feng, Claire Tipton, Julius N. De Castro, Vu N. Bach, Clariza Borile, Alexandria LaSalla, Hussain N. Odeh, Miranda Yeung, Rafael Garcia-Mata, Kam C. Yeung

**Affiliations:** 1 Department of Cancer Biology, College of Medicine and Life Sciences, University of Toledo, Health Science Campus, Toledo, OH 43614 USA; 2 Department of Biological Sciences, College of Natural Sciences, University of Toledo, Toledo, OH 43614 USA; 3 Present Address: Department of Plastic and Cosmetic Surgery, Nanfang Hospital, Southern Medical University, 1838 Guangzhou North Road, Guangzhou, 510515 Guangdong People’s Republic of China

**Keywords:** Biochemistry, Cancer, Cell biology, Genetics, Molecular biology, Cancer

## Abstract

Raf-1 kinase inhibitor protein was initially discovered as a physiological kinase inhibitor of the MAPK signaling pathway and was later shown to suppress cancer cell invasion and metastasis. Yet, the molecular mechanism through which RKIP executes its effects is not completely defined. RhoA has both a pro- and anti-metastatic cell-context dependent functions. Given that Rho GTPases primarily function on actin cytoskeleton dynamics and cell movement regulation, it is possible that one way RKIP hinders cancer cell invasion/metastasis is by targeting these proteins. Here we show that RKIP inhibits cancer cell invasion and metastasis by stimulating RhoA anti-tumorigenic functions. Mechanistically, RKIP activates RhoA in an Erk2 and GEF-H1 dependent manner to enhance E-cadherin membrane localization and inhibit CCL5 expression.

## Introduction

RKIP was identified and named by our lab as a physiologically relevant inhibitor of the Raf-MEK-Erk signaling cascade^1,2^. It has since been found to modulate several additional signaling pathways including NF-κB, keap1/nrf2, STAT3, and GSK. Since these signaling pathways play an important role in cancer metastasis, it was anticipated that RKIP might function as a metastasis suppressor. Indeed, the expression of RKIP is significantly decreased in many types of cancers and further reduced in their distant metastases. Significantly, restoration of RKIP expression inhibits prostate and breast cancer metastasis. In addition, loss of RKIP expression has been an important indicator of poor prognosis in these same types of malignancies.

Metastasis is defined as the complex multi-step formation of progressively growing secondary tumors in distant organs. RKIP has been shown to suppress tumor angiogenesis, local invasion, intravasation, bone and lung metastasis in murine models^3–6^. Several molecular mechanisms have been reported to explain RKIP’s role in metastasis, yet a comprehensive representation is still lacking. In our present study, we explore the effect of RKIP on Rho small GTPases. Being uniquely activated/deactivated by GTP/GDP binding, Rho proteins are not traditionally regulated at transcription and translation levels, making it challenging to identify Rho signaling mediators through conventional methods. In early studies, RhoA GTPases were reported to be overexpressed in cancers and suggested an oncogenic role in tumor progression^7,8^. Yet recent high-throughput sequencing efforts have identified loss-of-function mutations in *RhoA*^9–12^, and negative RhoA regulators, RhoGAPs, as oncogenes suggesting a tumor suppressive role of RhoA^13,14^. RhoA is a protein of multiple cellular regulatory functions that can either enhance or stymie cancer progression and metastasis. It is possible that the functions of RhoA in cancer are cell-context dependent. Here we demonstrate that RKIP specifically activates RhoA GTPase in an Erk2- and GEFH1-dependent manner. We showed that RKIP suppressed breast cancer cells invasion and metastasis by stimulating the anti-tumor functions of RhoA.

A possible mechanism of how RhoA mediates its tumor suppressive activity is through stabilizing the E-cadherin (E-cad) containing adherens junctions (AJs)^15^. AJs are protein complexes that occur at cell–cell junctions in epithelial and endothelial tissues. AJs initiate, stabilize cell–cell adhesion, regulate the actin cytoskeleton, intracellular signaling and transcription^16^. E-cad protein is the essential component in AJs, and loss of E-cadherin is associated with poorly differentiated breast tumors and a poor prognosis^17,18^. Inactivation of E-cad in mammary glands, impair lactation and accelerate tumor invasion and metastasis after loss of tumor suppressor gene p53^19^. Here we show that the RhoA-mediated E-cad membrane localization positively correlates with RKIP’s anti-invasive, and -metastatic functions. In addition, we showed that activation of RhoA was the cause of the previously observed reduced CCL5 expression and macrophages infiltration in tumors with restored RKIP expression, revealing another possible mechanism used by RhoA to inhibit metastasis.

## Results

### RKIP activates RhoA to suppress breast cancer cell invasion

**Figure 1:**
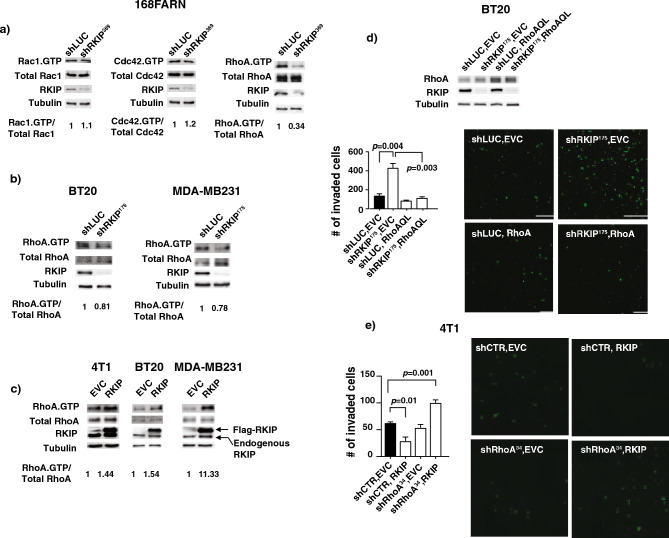
RKIP activates RhoA to suppress breast cancer cell invasion. (**a**) Representative western blots with the indicated Abs of GTPase pull-down assays with lysates prepared from control knockdown (shLUC) or RKIP knockdown (shRKIP^369^) 168 FARN cells as indicated. Numbers are shown for quantified active GTPase bands normalized with total protein. (**b**) Representative western blots of GTPase pull-down assays with lysates prepared from control knockdown (shLUC) or RKIP knockdown (shRKIP^175^) BT20 (left) and MDA-MB231 (right) cells as indicated. Numbers are shown for quantified active RhoA bands normalized with total protein. (**c**) Representative western blots of GTPase pull-down assays with lysates prepared from control (EVC) or RKIP expressing (RKIP) 4T1(left), BT20 (middle) and MDA-MB231 (right) as indicated. Numbers are shown for quantified active RhoA bands normalized with total protein. (**d**) (Top) Representative western blots of lysates prepared from BT20 cells with different combinations of controls, RKIP knockdown (shRKIP^175^) and RhoAQL expression as indicated. (Left, bottom) Representative results of invasion assays with same set of cells showing number of invaded cells through Matrigel in each indicated BT20 cell lines (mean ± SE). (Right, bottom) Representative images of the stained invaded cells in indicated cell lines shown at the bottom left panel. (**e**) (Left) Number of invaded 4T1 cells with different combinations of controls, RhoA knockdown (shRhoA^34^) and RKIP expression as indicated through Matrigel (mean ± SE). (Right) Representative images of the stained invaded cells in indicated cell lines shown in the left panel. Unpaired Student's t-test (two-tailed) was used for all analyses with *p* < 0.05 considered significant.

RKIP suppresses breast cancer cells invasion in vitro and metastasis in vivo. In an effort to identify signaling pathways that are targeted by RKIP for invasion/metastasis inhibition, we studied genes that were differentially affected by altered RKIP expression and identified chemokines and matrix metalloproteinases gene families as two possible RKIP targets^4,6^. Nonetheless, microarray approach only detects differences in transcription initiation and mRNA stability. Differences in certain signaling pathways like Rho GTPase signaling, which are mainly regulated at the post-translational level, will not be detected by such approach. Rho GTPase proteins exist in two conformations, GTP-bound active, and the GDP-bound inactive forms, and the switch between two conformations is regulated by GEF and GAP regulatory proteins. As a major signal transducer in the actin and tubulin cytoskeletal regulation, Rho GTPases affect several physiological processes required for cell migration and invasion. Therefore, RKIP might inhibit cell invasion and metastasis through Rho GTPases. To investigate the role of RKIP in Rho GTPase signaling, we stably downregulated the RKIP expression by viral transduction of specific shRNAs and measured the effect of RKIP knockdown on major Rho proteins activation in 168FARN mouse breast cancer cells. While reduced RKIP expression significantly reduced the activity of RhoA, it did not significantly affect the activation of Rac1 or Cdc42 GTPases (Fig. 1a). To eliminate possible off-target effect of shRNA and validate RKIP’s effect on RhoA activation is not species and cell lines specific, we generated additional RKIP knockdown human breast cancer cells with a different RKIP specific shRNA. Significantly we observed a similar effect on RhoA activities upon reduction of RKIP expression in two additional human breast cancer cells (Fig. 1b). The observed effects deem physiological as increased expression of RKIP resulted in an opposite effect (Fig. 1c). While there was an effect on the GTPase activity of RhoA in breast cancer cells with altered RKIP expression, we did not observe any difference in the RhoA transcripts as measured by qt-RT-PCR or DNA microarray analysis (data not shown).

**Figure 2:**
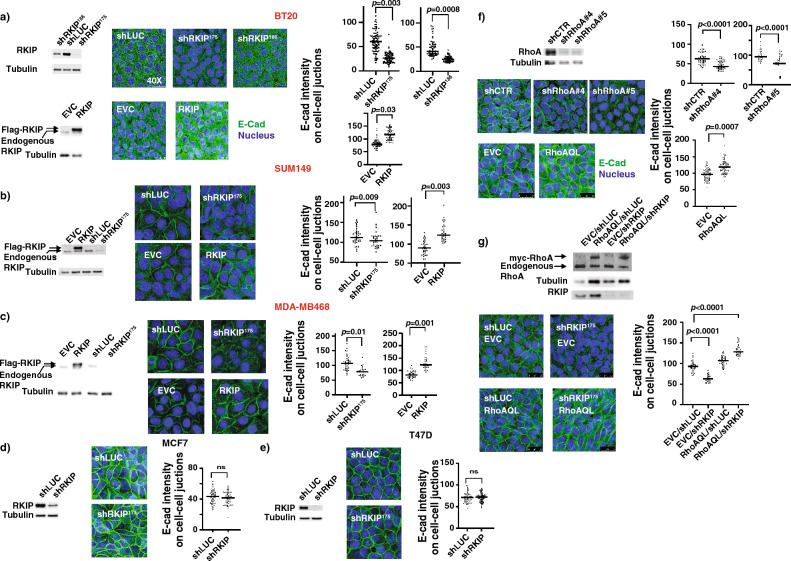
RKIP functions through downstream RhoA to promote E-cadherin (E-cad) localization to cell-to-cell junctions. (**a**) (Left) Representative western blots of lysates prepared from control knockdown (shLUC), two different RKIP knockdowns (shRKIP^175/186^), empty vector control, or RKIP expressing BT20 cells. (Middle) Representative images of immunofluorescent staining with E-cad Ab and DRAQ5 of the same set of BT20 cells shown in the left panel. (Right) E-cad intensity quantification on cell–cell junctions of immunofluorescent images shown in the middle panel using MetaMorph analysis software. (**b**) (Left) Representative western blots of lysates prepared from control knockdown (shLUC), RKIP knockdowns (shRKIP^175^), empty vector control (EVC), or RKIP expressing SUM149 cells. (Middle) Representative images of immunofluorescent staining with E-cad Ab and DRAQ5 of the same set of SUM149 cells shown in the left panel. (Right) E-cad intensity quantification on cell–cell junctions of immunofluorescent images shown in the middle panel using MetaMorph analysis software. (**c**) (Left) Representative western blots of lysates prepared from control knockdown (shLUC), RKIP knockdowns (shRKIP^175^), empty vector control (EVC), or RKIP expressing MDA-MB468 cells. (Middle) Representative images of immunofluorescent staining with E-cad Ab and DRAQ5 (Nucleus) of the same set of MDA-MB468 cells shown in the left panel. (Right) E-cad intensity quantification on cell–cell junctions of immunofluorescent images shown in the middle panel using MetaMorph analysis software. (**d**) (Left) Representative western blots of lysates prepared from control knockdown (shLUC), or RKIP knockdowns (shRKIP^175^) MCF7 cells. (Middle) Representative images of immunofluorescent staining with E-cad Ab and DRAQ5 of the same set of MCF7cells shown in the left panel. (Right) E-cad intensity quantification on cell–cell junctions of immunofluorescent images shown in the middle panel using MetaMorph analysis software. (**e**) (Left) Representative western blots of lysates prepared from control knockdown (shLUC), or RKIP knockdowns (shRKIP^175^) T47D cells. (Middle) Representative images of immunofluorescent staining with E-cad Ab and DRAQ5 of the same set of T47D cells shown in the left panel. (Right) E-cad intensity quantification on cell–cell junctions of immunofluorescent images shown in the middle panel using MetaMorph analysis software. (**f**) (Left, top) Representative western blots of lysates prepared from control knockdown (shCTR), two different RhoA knockdowns (shRhoA#4/#5), empty vector control, or RhoAQL expressing BT20 cells. (Left, bottom) Representative images of immunofluorescent staining with E-cad Ab and DRAQ5 of the same set of BT20 cells shown in the left top panel. (Right) E-cad intensity quantification on cell–cell junctions of immunofluorescent images shown in the left bottom panels using MetaMorph analysis software. (**g**) (Left, top) Representative western blots of lysates prepared from BT20 cells with different combinations of controls, RKIP knockdown (shRKIP^175^) and RhoAQL expression as indicated. (Left, bottom) Representative images of immunofluorescent staining with E-cad Ab and DRAQ5 of the same set of BT20 cells shown in the left top panel. (Right) E-cad intensity quantification on cell–cell junctions of immunofluorescent images shown in the left bottom panels using MetaMorph analysis software. Unpaired Student's t-test (two-tailed) was used for all analyses with *p* < 0.05 considered significant. *ns* not significant.

We have previously shown that both RKIP and RhoA are negative regulators of breast cancer cell invasion^20,21^. Knocking down of RKIP expression increased the capacity of BT20 breast cancer cells to invade in vitro (Fig. 1d). Here we show that reduced RKIP expression decreases RhoA GTPase activity. It is possible that the decrease RhoA activity is the cause of the increased invasiveness of the RKIP knockdown cells. To examine this possibility, we expressed sub-optimal amount of a constitutively active RhoA variant Q63L in RKIP knockdown BT20 cells. As expected, sub-optimal expression of RhoAQ63L had insignificant effect on BT20 cells invasion (compare column 3 with 1 in Fig. 2a). However, co-expression of the same amount of RhoAQ63L completely reversed the effect on cells invasion owing to the knocking down of RKIP expression in BT20 cells (compare column 4 with 1 & 2 in Fig. 1d). Our results therefore suggested a possible involvement of RhoA in RKIP-mediated suppression of breast cancer cell invasion. In line with this reasoning, sub-optimal reduction in RhoA expression is also sufficient to reverse the effect on invasion resulted from ectopic expression of RKIP in 4T1 cells (Fig. 1e).

### RKIP suppresses breast cancer cell invasion through RhoA-mediated regulation of E-cadherin

RhoA has been previously reported to be required for the positioning E-cadherin (E-cad) in cell–cell junctions in epithelial cells^22^. E-cad is an adhesion protein that connects epithelial cells together and its loss in expression or mis-localization enhances cell migration/invasion and metastasis^22–24^. It is therefore possible that RKIP suppresses invasion/metastasis by regulating E-cad expression or localization through RhoA. To investigate this possibility, we examined the effects of altered RKIP expression on E-cadherin expression and localization in BT20 cells by immunofluorescent staining. Consistent with the positively regulatory role of RKIP on RhoA activity (Fig. 1), downregulation of RKIP expression by specific shRNAs showed a dose-dependent loss while ectopic expression of RKIP had an increase in the expression of E-cad in BT20 cells (Fig. 2a). BT20 is a triple-negative epithelial-like subset of breast cancer cell line. The effect of RKIP on E-cad expression was not BT20 cell line specific. We also observed similar effect in other triple-negative epithelial cell lines SUM149, and MDA-MB468 (Fig. 2b,c). However, RKIP has no observable role in regulating Ecad expression in non-triple negative MCF7, and T47D cells (Fig. 2d,e). Additionally, RKIP might have a positive effect on the expression of the *E-cad* gene as well, since stable RKIP knockdown decreased the *E-cad* gene fold expression and the E-cad protein expression (Supp Fig. 1). As expected, stable downregulation of RhoA expression by specific shRNA caused a significant reduction in the membrane E-cad expression (Fig. 2f) and with an opposite effect upon ectopic expression of constitutively active RhoA variant (Fig. 2f). It is of interest to note that unlike RKIP, altered expression of RhoA has no observable effects on the E-cad protein expression as detected by the western blotting (Supp Fig. 1).

In order to determine whether RKIP’s ability to regulate E-cad localization depends on the downstream RhoA, BT20 cells grown on coverslips were immunostained for E-cadherin. Upon stable knockdown of RKIP, E-cadherin was less localized on the membrane, and this effect could be rescued by the stable co-expression of RhoAQL in RKIP knockdown cells. (Fig. 2f) Collectively, these results indicated that, in BT20 cells, RKIP functions through downstream RhoA to promote E-cadherin localization to the cell-to-cell junctions.

**Figure 3:**
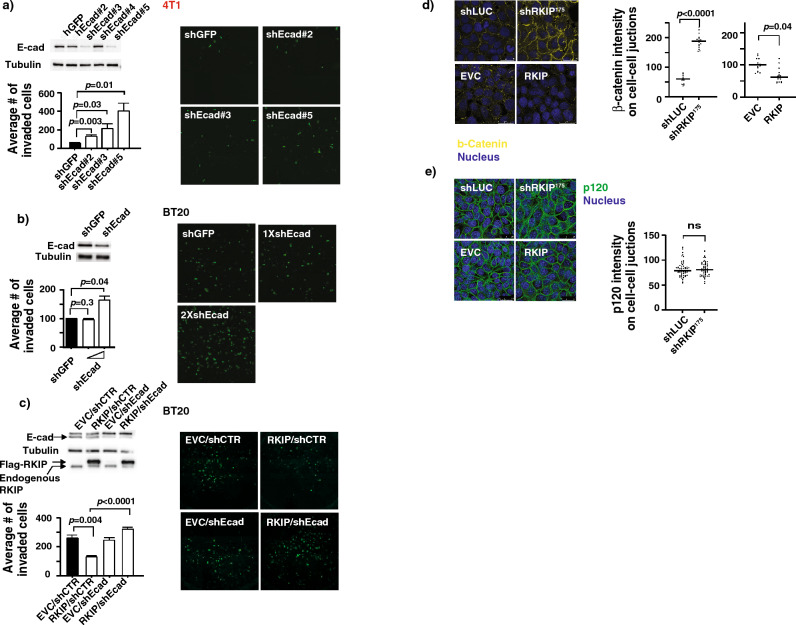
RKIP-mediated inhibition of cell invasion depends on the E-cadherin (E-cad) cell–cell junction localization. (**a**) (Top left panel) Representative western blots of lysates prepared from control knockdown (shGFP), or four different E-cad knockdowns (shEcad#2–5) 4T1 cells. (Bottom left panel) Representative results of invasion assays with same set of cells showing number of invaded cells through Matrigel in each indicated 4T1 cell lines (mean ± SE). (Right panel) Representative images of the stained invaded cells in indicated cell lines shown in the bottom left panel. (**b**) (Top left panel) Representative western blots of lysates prepared from control knockdown (shGFP), or E-cad knockdowns BT20 cells. (Bottom left panel) Representative results of invasion assays with shGFP control knockdown or titrated E-cad knockdown showing number of invaded cells through Matrigel in each indicated BT20 cell lines (mean ± SE). (Right panel) Representative images of the stained invaded cells in indicated cell lines shown in the bottom left panel. (**c**) (Top left panel) Representative western blots of lysates prepared from BT20 cells with different combinations of controls, E-cad knockdown (shEcad) and RKIP expression as indicated. (Bottom left panel) Representative results of invasion assays with same set of cells showing number of invaded cells through Matrigel in each indicated BT20 cell lines (mean ± SE). (Right panel) Representative images of the stained invaded cells in indicated cell lines shown at the bottom left panel. (**d**) (Left panel) Representative western blots of lysates prepared from BT20 cells with different combinations of controls, RKIP knockdown (shRKIP^175^) and RhoAQL expression as indicated. (Left, bottom) Representative images of immunofluorescent staining with β-Catenin Ab and DRAQ5 (Nucleus) of BT20 cells with control knockdown (shLUC), RKIP knockdown (shRKIP), empty vector control (EVC) or RKIP expression. (right panel) β-Catenin intensity quantification on cell–cell junctions of immunofluorescent images shown in the left panel using MetaMorph analysis software. Representative images of immunofluorescent staining with p120 Ab and DRAQ5 (Nucleus) of BT20 cells with control knockdown (shLUC), RKIP knockdown (shRKIP), empty vector control (EVC) or RKIP expression. (Right panel) p120 intensity quantification on cell–cell junctions of immunofluorescent images shown in the left panel using MetaMorph analysis software. Unpaired Student's t-test (two-tailed) was used for all analyses with *p* < 0.05 considered significant. *ns* not significant.

To determine if the change in E-cad expression levels is the cause of RKIP-mediated suppression of breast cancer cells invasion, we examine the effect of sub-optimal reduction of E-cad expression on the invasion of RKIP ectopically expressed BT20 cells. As expected, downregulation of E-cad expression increased invasion in a dose-dependent manner (Fig. 3a,b). Ectopic expression of RKIP suppressed BT20 cell invasion, while concurrent sub-optimal knockdown of E-cadherin expression reversed the inhibitory effect on invasion due to the expression of RKIP indicating the increase in E-cad expression is required for the RKIP-mediated suppression of breast cancer cell in vitro invasion (Fig. 3c).

E-cad can exist as a component of protein complexes called adherens junctions (AJs) located in the cell–cell junctions of epithelial tissues. In addition to E-cad, the other components of AJs include α-, β-, δ- (also called p120) and γ- (also called plakoglobin) catenins. E-cadherin, the major functional unit in the AJs, has an extracellular region with five cadherin domains that interact homophilically with cadherin domains in neighboring cells, and an intracellular cytoplasmic tail that directly binds p120 catenin and β-catenin through conserved binding sites. α-catenin binds to AJs indirectly through β-catenin linking AJs to the actin cytoskeleton^25^. To determine if RKIP expression has an effect on other components of the AJs we examine the expression and localization of β-catenin and p120 in BT20 cells with altered RKIP expression by confocal microscopy after immunostaining with specific antibodies. While we did not detect any effect on p120, our results support a negative regulatory role of RKIP on β-catenin expression (Fig. 3d,e.).

### RKIP activates RhoA through Erk2 and GEFH1

**Figure 4:**
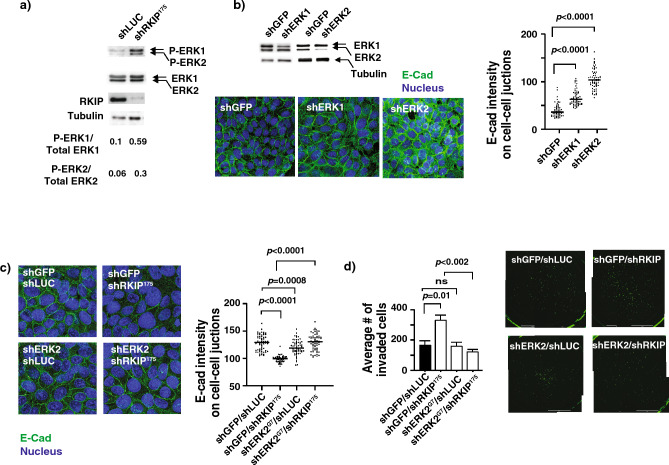
RKIP regulates E-cadherin (E-cad) and subsequent cell invasion through ERK2. (**a**) Representative western blots with lysates prepared from control knockdown (shLUC) or RKIP knockdown (shRKIP) BT20 as indicated. Numbers are shown for quantified phosphorylated ERK1 or ERK2 bands normalized with total ERK protein. (**b**) (Top left panel) Representative western blots of lysates prepared from control knockdown (shGFP), ERK1 (shERK1) or ERK2 (shERK2) BT20 cells. (Bottom left panel) Representative images of immunofluorescent staining with E-cad Ab and DRAQ5 of the same set of BT20 cells shown in the left top panel. (Right panel) E-cad intensity quantification on cell–cell junctions of immunofluorescent images shown in the left bottom panels using MetaMorph analysis software. (**c**) (Left panel) Representative images of immunofluorescent staining with E-cad Ab and DRAQ5 of BT20 cells with different combinations of control knockdown, RKIP knockdown (shRKIP) or ERK2 knockdown (shERK2). right) E-cad intensity quantification on cell–cell junctions of immunofluorescent images shown in the left bottom panels using MetaMorph analysis software. (**d**) (Left panel) Representative results of invasion assays with same set of cells used in (**c**) showing number of invaded cells through Matrigel in each indicated BT20 cell lines (mean ± SE). (Right panel) Representative images of the stained invaded cells in indicated cell lines shown at the left panel. Unpaired Student's t-test (two-tailed) was used for all analyses with *p* < 0.05 considered significant. *ns* not significant.

In an attempt to understand the intermediate signal transducers between RKIP and RhoA in regulating E-cadherin localization and breast cancer cell invasion/metastasis, we examined the effect of Erk1/2 on E-cadherin localization in BT20 cells. ERK is a downstream kinase of the Raf-MEK-Erk pathway where RKIP acts as a negative regulator by binding to Raf-1 and inhibiting the kinase activity of Raf-1 kinase (Fig. 4a). We stably downregulated the Erk 1/2 expression by lentiviral transduction of specific shRNAs for Erk 1 and Erk 2 and stained these cells with E-cadherin antibody. As previously reported^26^, Erk1 knockdown did not have a substantial effect on the E-cad expression and the membrane localization in BT20, while specific Erk2 knockdown significantly elevated the membrane E-cadherin localization compared to the knockdown control cells, indicating an Erk2-specific negative regulatory effect on membrane E-cadherin (Fig. 4b). To examine whether RKIP regulates E-cadherin and subsequent cell invasion through Erk2, we sub-optimally reduced the expression of Erk2 in BT20 RKIP knockdown cells and studied its effect on E-cadherin localization and cell invasion (Fig. 4c,d). In agreement with earlier results, reduced expression of RKIP in BT20 cells decreased the membrane E-cadherin localization and significantly increased their invasiveness. Importantly, concomitant downregulating expression of Erk2 in RKIP knockdown cells reversed the effect on E-cad localization and invasion due to the loss of RKIP revealing that RKIP regulates E-cadherin and subsequent cell invasion through Erk2 (Fig. 4c,d).

**Figure 5:**
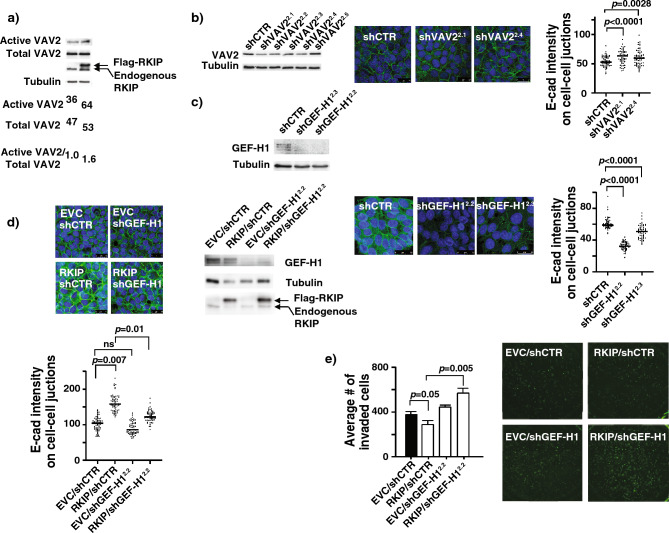
RKIP activates RhoA through GEF-H1. (**a**) Representative western blots of VAV2 GEF activity pull-down assays with lysates prepared from BT20 cells. Numbers are shown for quantified active VAV2 bands normalized with total VAV2 protein. (**b**) (Left panel) Representative western blots of lysates prepared from control knockdown (shCTR), or five different VAV2 knockdowns (shVAV2^2.1–2.5^) BT20 cells. (Middle panel) Representative images of immunofluorescent staining with E-cad Ab and DRAQ5 of the same set of BT20 cells shown in the left top panel. (Right panel) E-cad intensity quantification on cell–cell junctions of immunofluorescent images shown in the left bottom panels using MetaMorph analysis software. (**c**) (Left panel) Representative western blots of lysates prepared from control knockdown (shCTR), or two different GEF-H1 knockdowns (shGEF-H1^2.2–2.3^) BT20 cells. (Middle panel) Representative images of immunofluorescent staining with E-cad Ab and DRAQ5 of the same set of BT20 cells shown in the left top panel. (Right panel) E-cad intensity quantification on cell–cell junctions of immunofluorescent images shown in the left bottom panels using MetaMorph analysis software. (**d**) (Top left panel) Representative images of immunofluorescent staining with E-Cad Ab and DRAQ5 of BT20 cells with different combinations of control knockdown, GEF-H1 knockdown (shGEF-H1), empty vector control (EVC) or RKIP expression. (Bottom left panel) E-cad intensity quantification on cell–cell junctions of immunofluorescent images shown in the left bottom panels using MetaMorph analysis software. (Right panel) Representative western blots of lysates prepared from the same set of cells shown in the left panel. (**e**) (Left panel) Representative results of invasion assays with same set of cells used in (**d**) showing number of invaded cells through Matrigel in each indicated BT20 cell lines (mean ± SE). (Right panel) Representative images of the stained invaded cells in indicated cell lines shown at the left panel. Unpaired Student's t-test (two-tailed) was used for all analyses with *p* < 0.05 considered significant. *ns* not significant.

The GTPase activity of RhoA is primarily regulated by RhoGEF positive regulators and RhoGAP negative regulators. Vastly dynamic spatiotemporal regulation through these regulators determines the RhoA-mediated downstream signaling effects. Erk has been previously reported to inhibit RhoA activation through negatively regulating GEFH1^27^. Another RhoGEF, VAV2, has been reported to activate RhoA downstream of growth factor receptor signaling^28^. Moreover, VAV2 gets activated upon the expression of RKIP in BT20 cells (Fig. 5a). Consequently, we examined the effect of GEFH1 and VAV2 knockdown on E-cadherin membrane localization by confocal microscopy after immunofluorescent staining. While knocking down of VAV2 expression slightly increased expression of membrane localized E-cad, reduced GEFH1 expression phenocopied effects of reduced RKIP expression on membrane E-cadherin expression suggesting GEFH1 as one of the intermediate signal transducers between RKIP and RhoA (Fig. 5b,c). To examine if RKIP regulates E-cadherin and subsequent cell invasion through GEFH1, we studied the effect of sub-optimal reduced expression of GEF-H1 in ectopic RKIP expressing cells on E-cad localization and the cell invasion (Fig. 5d,e). As expected, RKIP expression increased the membrane E-cad and repressed cell invasion. In line with the thought that GEF-H1 may be involved in the activation of RhoA by RKIP, knockdown of GEF-H1 reversed the E-cadherin localization and restored their invasiveness in RKIP expressed BT20 cells (Fig. 5d,e). Collectively, these results suggested the existence of a signal transduction from RKIP to breast cancer invasion/metastasis through Erk2, GEFH1, RhoA and E-cad.

### Increased RhoA expression is required for RKIP-mediated suppression of breast cancer metastasis in a murine allograft model

**Figure 6:**
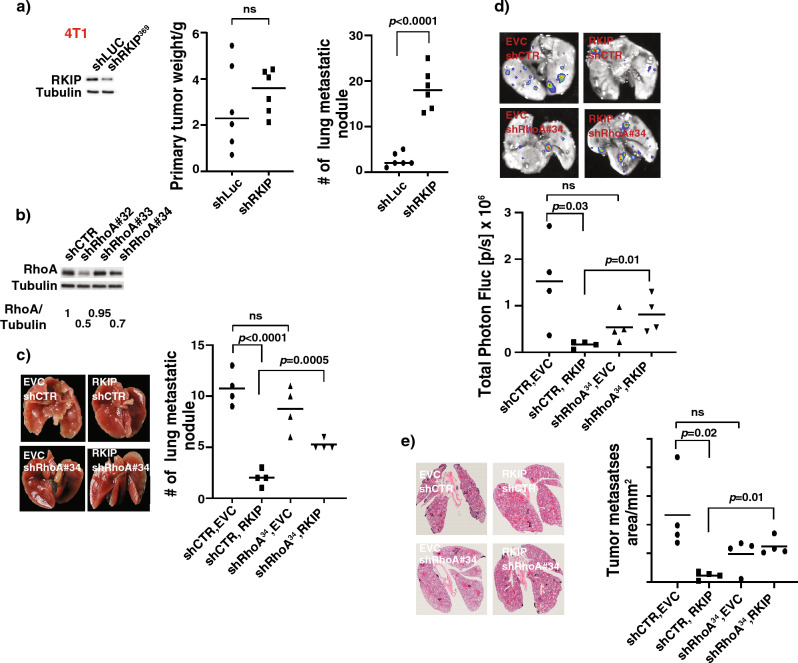
Increased RhoA expression is required for RKIP-mediated suppression of breast cancer metastasis in a murine allograft model. (**a**) (Left panel) Representative western blots of lysates prepared from control knockdown (shLUC), or RKIP knockdown (shRKIP^369^) 4T1 cells. (Middle panel) Weight of primary tumors harvested from Balb/c mice 30 days after orthotopical implantation with the indicated 4T1 cells shown in the left panel. (Right panel) Number of lung metastatic nodules recorded in Balb/c mice 30 days after orthotopical implantation with the indicated 4T1 cells shown in the left panel. n = 6. (**b**) Representative western blots of lysates prepared from control knockdown (shCTR), or three different RhoA knockdowns (shrhoA#32-34) 4T1 gfp-luc cells. The data shown in Fig. 6(**b**) was used previously in another article (Fig. 2d)^20^. The duplicated data was used to confirm the RhoA expression was successfully knocked down in a breast cancer cell line that was used in both publications. (**c**) (Left panel) Representative ex vivo color images of lungs harvested from mice orthotopically injected with the indicated 4T1 gfp-luc cells showed in (**b**), (Right panel) Quantification of lung metastatic nodules (mean ± SE) detected in lungs harvested from mice orthotopically injected with the indicated 4T1 gfp-luc cells shown in (**b**). n = 4. (**d**) (Top panel) Representative ex vivo BLI images of lungs harvested from mice orthotopically injected with the indicated 4T1 gfp-luc cells shown in (**b**), (Bottom panel) Photon flux quantification of (mean ± SE) BLI images shown in the top panel. n = 4. (**e**) (Left) Representative hematoxylin and eosin (H&E) stained cross-sections of lungs harvested from mice orthotopically injected with the indicated 4T1 gfp-luc cells showed in (**b**) showing metastases demarcated from normal tissues with black solid line. (Right panel) total tumor metastases area calculated from the H&E sections shown above. n = 4. Unpaired Student's t-test (two-tailed) was used for all analyses with *p* < 0.05 considered significant. *ns* not significant.

Ectopic expression of RKIP suppresses the proclivity of breast cancer cells to metastasize to lungs in orthotopic mouse models^6^. We reasoned that we should detect the opposite effect if the observed effect is physiologically relevant. Indeed, while there were no significantly differences in primary tumor weights, we detected a significantly elevated metastatic burden in lungs of mice orthotopically transplanted with stably RKIP knockdown mouse 4T1 breast cancer cells when compared with controls (Fig. 6a). Since we detected a similar effect when RhoA was knockdown^20^, it is possible that RKIP and RhoA are in the same pathway that regulates breast cancer cells lung metastasis. To address this possibility, we sub-optimally knockdown the expression of RhoA in RKIP ectopically expressed 4T1 for orthotopic cancer cells transplantation assay (Fig. 6b–d). All injected mice developed primary tumors. However, we detected no significant differences in size or growth rate of the primary tumors as measured by whole-animal bioluminescence imaging and quantifying the expression of proliferation antigen Ki67 at 30 days after implantation (Supp Fig. 2). On the contrary, ex-vivo bioluminescence imaging (BLI) of freshly harvested lungs revealed a significant difference in lung BLI signals between groups suggesting differences in metastatic burden (Fig. 6d). This was further substantiated by counting the number of macro nodules on the lung surface and quantifying the tumor metastases area in an H&E-stained lung section (Fig. 6c–e). As expected, RKIP expressing 4T1 cells-injected mice showed the least metastatic burden. Yet, the concurrent RhoA knockdown in RKIP-expressed cells, caused a significant enhancement in the metastatic signal in the mice compared to the RKIP expressing 4T1 cells-injected mice (Fig. 6c–e). Collectively, these results suggested that an increase in RhoA expression is required for RKIP-mediated suppression of breast cancer cell invasion and metastasis.

### RKIP suppresses breast cancer metastasis partially through downstream RhoA-regulated mechanisms

**Figure 7:**
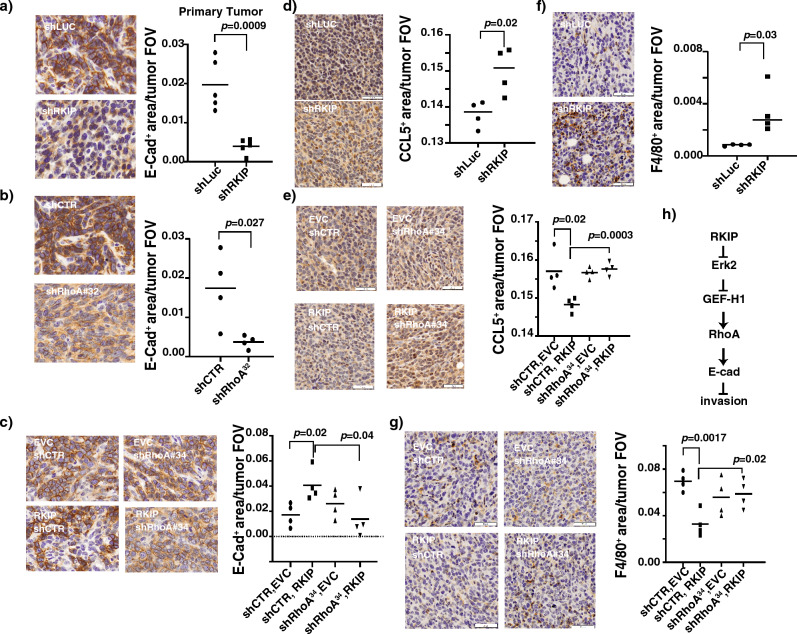
RKIP suppresses breast cancer metastasis in part through downstream RhoA-regulated mechanisms. (**a**) (Right panel) Representative immunohistochemical (IHC) E-cad Ab staining images of breast primary tumor sections of mice orthotopically injected with control knockdown (shLUC) or RKIP (shRKIP) knockdown 4T1 gfp-luc cells. (Left panel). Quantification of areas per tumor field of view (FOV) stained positive in the right panel for E-cad. n = 4. (**b**) (Right panel) Representative immunohistochemical (IHC) E-cad Ab staining images of breast primary tumor sections of mice orthotopically injected with control knockdown (shCTR) or RhoA (shRhoA#32) knockdown 4T1 gfp-luc cells. (Left panel). Quantification of areas per tumor field of view (FOV) stained positive in the right panel for E-cad. n = 4. (**c**) (Right panel) Representative immunohistochemical (IHC) E-cad Ab staining images of breast primary tumor sections of mice orthotopically injected with 4T1 gfp-luc cells with different combinations of control knockdown, RhoA knockdown (shRhoA#34), empty vector control (EVC) or RKIP expression. (Left panel). Quantification of areas per tumor field of view (FOV) stained positive in the right panel for E-cad. n = 4. (**d**) (Right panel) Representative immunohistochemical (IHC) CCL5 Ab staining images of breast primary tumor sections of mice orthotopically injected with control knockdown (shLUC) or RKIP (shRKIP) knockdown 4T1 gfp-luc cells. (Left panel). Quantification of areas per tumor field of view (FOV) stained positive in the right panel for CCL5. n = 4. (**e**) (Right panel) Representative immunohistochemical (IHC) CCL5 Ab staining images of breast primary tumor sections of mice orthotopically injected with 4T1 gfp-luc cells with different combinations of control knockdown, RhoA knockdown (shRhoA#34), empty vector control (EVC) or RKIP expression. (Left panel). Quantification of areas per tumor field of view (FOV) stained positive in the right panel for CCL5. n = 4. (**f**) (Right panel) Representative immunohistochemical (IHC) F4/80 Ab staining images of breast primary tumor sections of mice orthotopically injected with control knockdown (shLUC) or RKIP (shRKIP) knockdown 4T1 gfp-luc cells. (Left panel). Quantification of areas per tumor field of view (FOV) stained positive in the right panel for F4/80. n = 4. (**g**) (Right panel) Representative immunohistochemical (IHC) F4/80 Ab staining images of breast primary tumor sections of mice orthotopically injected with 4T1 gfp-luc cells with different combinations of control knockdown, RhoA knockdown (shRhoA#34), empty vector control (EVC) or RKIP expression. (Left panel). Quantification of areas per tumor field of view (FOV) stained positive in the right panel for F4/80. n = 4. Unpaired Student's t-test (two-tailed) was used for all analyses with *p* < 0.05 considered significant. (**h**) A schematic depicts the mechanism through which RKIP activates E-cad to suppress cancer cells invasion.

E-cadherin is a well-established tumor metastasis suppressor. Here we show that RKIP increases E-cad expression through RhoA and the increased E-cad expression is the cause of RKIP-mediated inhibition of in vitro breast cancer cell invasion. It is therefore possible that RKIP mitigates breast cancer metastasis partly by enhancing E-cadherin junctional localization and stabilization of adherens junctions through RhoA in primary tumors. To entertain this possibility, we examined the E-cadherin expression in primary tumors harvested from orthotopically injected mice with stable RKIP or RhoA knockdown 4T1 cells. Consistent with results generated with cell-based studies, the total E-cadherin expression of RKIP and RhoA knockdown primary tumors was significantly reduced when compared with the control knockdown tumors (Fig. 7a,b). Higher magnified immunochemical stained images revealed the mostly membrane expression pattern for E-cadherin observed in knockdown control tumors was vanished in RhoA or RKIP knockdown tumors (Fig. 7a,b). Collectively, these observations established a physiologically relevant correlation between RKIP/RhoA expression and membrane E-cadherin expression and localization, and the metastatic potential of 4T1 primary breast tumors.

To examine if the regulation of E-cad expression by RKIP is RhoA dependent, we quantitate E-cadherin expression in primary tumors from mice injected with ectopic RKIP expressed 4T1 cells with RhoA sub-optimal knockdown. Contrary to RKIP knockdown tumor, ectopic RKIP expressed primary tumors showed a significantly higher membrane E-cadherin expression. While sub-optimal RhoA knockdown had no significant effect on E-cad when compared with control, knocking down expression of RhoA in RKIP expressing 4T1 was sufficient to reverse the effect on E-cad expression resulted from ectopic RKIP expression (Fig. 7c). Our results, therefore suggest that RKIP enhances the positioning of E-cad in cell–cell junction through RhoA in primary tumors.

We and others have shown that ectopic expression of RKIP partially suppresses breast cancer lung metastasis and F4/80^+^ tumor macrophage infiltration by inhibiting CCL5 expression in orthotopic mouse models^6,29^. We reasoned that if the observed effect is physiologically relevant, we should detect the opposite effect when RKIP expression is inhibited. Indeed, we detected a significantly increased tumor CCL5 expression and F4/80^+^ macrophage infiltration in the primary tumors when stably RKIP knockdown mouse 4T1 breast cancer cells were transplanted orthotopically into mice (Fig. 7d,e). Since we detected a similar effect when RhoA was knockdown^20^, it is possible that the effect of altered RKIP expression on CCL5 and macrophage infiltration is RhoA dependent. To entertain this possibility, we quantitated the expression levels of CCL5 and F4/80^+^ macrophage infiltration by IHC staining of primary tumors harvested from mice injected with sub-optimal RhoA knockdown RKIP-expressed 4T1 cells. Consistent with previous results, RKIP-expressed tumors had significantly reduced CCL5 expression and F4/80 staining when compared with control tumors (Fig. 7f). In support of our proposition that RKIP regulates CCL5 expression through RhoA, knocking down the expression of RhoA was sufficient to reverse the effects on CCL5 and F4/80 expression in RKIP-expressing 4T1 (Fig. 7g). Previously, we reported that RKIP inhibits F4/80^+^ macrophage infiltration by decreasing CCL5 expression in cancer cells^6^. Here we show that the RKIP-mediated inhibition of CCL5 expression and F4/80^+^ cells tumor infiltration can be reversed by silencing of of RhoA expression suggesting RKIP may regulate CCL5 expression through RhoA GTPases.

## Discussion

Conceptually RKIP can interfere directly with the metastasis cascade or indirectly by regulating the activity of single or multiple metastasis genes. Unbiased systematic transcriptome analyses have revealed that RKIP regulates the expression of multiple metastasis genes in interconnecting signaling pathways. However, the decrease in metastasis caused by RKIP expression is not fully restored by a gain-of-function in the RKIP targeted signaling pathways^4,6,29,30^, indicating that there are additional RKIP targets. Here, we report that RKIP also targets RhoA for post-translational regulation to inhibit metastasis.

RhoA is a member of the Rho family of GTPase and has a dual role in cancer cells invasion. It can have either a positive or negative role in cancer metastasis, but its mechanistic basis and operational logic is not well defined. We showed that RKIP stimulated the GTPase activity of RhoA to inhibit breast cancer cells invasion. Among the various effectors, mDia and ROCK are two best-described effectors that are important for RhoA-mediated regulation of cancer cell invasion/metastasis by stabilizing AJs, regulating myosin light chain 2 (MLC2) phosphorylation and focal adhesion dynamics^31^. AJs are multi-protein complexes that mediate homotypic cell adhesion in epithelial and endothelial tissues^16^. Membrane-spanning E-cadherin, the key component of the complex, mediates cell–cell contact through interacting with E-cad molecules on opposing cells^32,33^. RKIP positively regulates the expression levels of E-cad on cell–cell junctions through RhoA in epithelial like triple-negative breast cancer cells. Loss of E-cad expression compromises AJs integrity and facilitates cancer cells motility. Our results therefore suggest that RKIP hinders cancer cell invasion by stabilizing the AJs. At present we do not know which RhoA effector proteins are involved in the RhoA dependent effects of RKIP on E-cad expression in cell–cell junctions. It was reported that in luminal type breast cancer cells, active RhoA increased E-cad localization to cell–cell contacts by activating mDia1^34^. Active mDia presumably stabilizes the E-cad and catenin containing AJs through increased nucleation and elongation of actin filaments. However, similar effect of mDia on E-cad localization was not observed in triple negative epithelial like breast cancer cells used in our study suggesting a possible breast cancer cell type specific effect of mDia on E-cad expression (unpublished results). We also do not know if altered RKIP expression has any effects on other RhoA effectors and their downstream targets.

In addition to interacting with E-cad of adjacent cells through its extracellular domains, cytoplasmic domains of E-cad molecules interact directly with p120-catenin and β-catenin, and indirectly with α-catenin^25^ forming the core of AJs. The association of E-cad with catenin connects AJs to the actin and microtubule cytoskeleton^35^. It remains to be determined if altered RKIP expression affects the cytoskeleton structure in cancer cells. While there were no discernable differences in the expression levels of p120-catenin, we detected significant increased expression of β-catenin in cell–cell junction of RKIP knockdown cells. In light of the prior report showing the E-cad dependent membrane localization of β-catenin, our results with β-catenin expression in RKIP knockdown cells are unexpected^36^. However, our results were consistent with the previous report that RKIP positively regulated GSK-3β kinase in the canonical Wnt signaling pathway^37^. β-Catenin is a dual function protein. Beside regulating cell–cell adhesion, β-catenin also plays an important role in transcription regulation as part of the Wnt signaling pathway^38^. GSK-3β phosphorylates and targets β-catenin for ubiquitin-mediated degradation^38^. Therefore, it is conceivable that the decrease in GSK-3b activity in RKIP knockdown cell is the cause of the observed increase in β-catenin expression.

Presently we have not detected interaction between RKIP and RhoA suggesting RKIP may activate RhoA indirectly. We considered Erk as proximate putative downstream effector of RKIP in a pathway leading to the activation of RhoA and subsequently, E-cad for three reasons. First, the activation phosphorylation of Erk is inhibited by RKIP^39^. Second, Erk1/2 regulates RhoA activation by inhibiting RhoA activator, GEF-H1^40^. Finally, Erk2 has been reported to inhibit the expression of E-cad^26^. Our results support a model that RKIP increases expression levels of E-cad on cell–cell junctions by inhibiting Erk1/2 to impede cancer cells invasion. Erk can inhibit the E-cad expression transcriptionally through upregulation of transcription repressor ZEB1/2^26^. Alternatively, Erk can decrease the membrane localization of E-cad post-translationally by inactivating GEF-H1^40^. Although our study did not address the causal role of ZEB1/2 in RKIP-mediated regulation of E-cad expression, our results conclusively demonstrated a link between RKIP, Erk, GEF-H1, RhoA, and E-cad expression in triple negative basal epithelial-like breast cancer cell lines. Functionally our epistatic analysis with TNBC showed that RKIP suppresses cell invasion by upregulating the membrane localization of E-cad. It is of interest to note that the effect is breast cancer subtype specific as the knockdown of RKIP expression had no effect on E-cad localization in luminal breast cancer cells. Thus, our data implicate a breast cancer subtype-specific regulation of E-cad expression and membrane localization by RKIP.

We previously showed that inactivation of RhoA increased breast cancer cells metastasis in a mouse orthotopic transplantation model^20^. Our results showing the partial reversal of RKIP gain-of-function metastasis-suppression phenotype with a loss-of-function in RhoA indicate that RKIP interacts genetically with RhoA to regulate breast cancer metastasis. RKIP inhibits tumor angiogenesis, F4/80^+^ macrophage infiltration, and multiple steps of the metastasis cascade including dissemination into sentinel lymph node, intravasation, and colonization^4,6,29,30^. Our observations that RhoA knockdown tumors displayed some but not all of the phenotypes in tumors with altered RKIP expression is in accordance with the findings that RKIP has multiple downstream effector targets. Earlier, we identified that RKIP inhibits tumor angiogenesis, F4/80^+^ macrophage infiltration, and lung metastasis by downregulating CCL5 expression in cancer cells^6^. The mechanism of how RKIP mitigates CCL5 expression is not known. Here we show that downregulation of RhoA expression rescued the RKIP-mediated inhibition of CCL5 expression and F4/80^+^ cells tumor infiltration. Our results therefore suggested that RKIP may inhibit CCL5 through activation of RhoA GTPases.

In addition to having a regulatory role in CCL5 expression, RhoA also increased E-cad TNBC membrane localization in our cell-based in vitro study. Similarly, we demonstrated a positive correlation between RKIP expression, membrane localization and expression of E-cad in primary breast tumors, and a subsequent negative correlation with their metastatic potential with the 4T1 orthotopic murine model of triple negative basal breast cancer. Loss of E-cadherin is a hallmark of epithelial-to-mesenchymal transition (EMT), a highly coordinated cell biological program that activated during tumor malignant progression, invasion and metastasis^19^.

Our study established the causal role of E-cad in RKIP-mediated inhibition of invasion in vitro. However, it remains to be determined if downregulation of E-cad can reverse some of the phenotypes revealed in breast tumor with restored expression of RKIP. In summary, the present study identified a physiological mechanism where RKIP suppresses breast cancer lung metastasis through Erk, GEFH1, RhoA, and E-cad.

## Methods

### Cell lines and reagents

4T1, T47D, MCF7, BT20, MDA-MB468 and MDA-MB231 breast cancer cell lines were cultured as described^21^. SUM149 human breast cancer cell lines were cultured in Ham’s F12 medium with 5% FBS) and 1% penicillin–streptomycin with supplement of 5 μg/ml insulin and 1 μg/ml hydroxycortisol. 4T1 cells were kindly provided by Dr. Fred Miller (Karmanos Cancer Institute, MI). SUM149 cells were kindly provided by Dr. Steve Ethier (SLKBase). MDA-MB468 was a gift from Dr. Mahasin Osman of the University of Toledo. T47D, MCF7, BT20, and MDA-MB231 cells were purchased from ATCC. The antibodies for E-cadherin #3195, Cdc42 #2466, β-catenin #8480, GEFH1 #4145, VAV2 #2848 and RhoA were obtained from Cell Signaling Technology, MA, USA. The use of antibodies for F4/80, tubulin and CCL5 was described previously^6^. Antibodies for p120 is a kind gift of Raman Dayanidhi of the University of Toledo. The use of shRNAs for specific expression silencing of RKIP expression in breast cancer cells was previously described^41^. The lentiviral vectors for shRNA against mouse RhoA and scrambled siRNA control used for stable cell infection were obtained from GeneCopeia, Inc.^20^. The lentiviral vectors for shRNA against Erk1, Erk2 or were obtained from John Blenis of Cornell University. pLKO.1 shRNAs for human RhoA (#1 TRCN0000047708, #2 TRCN0000047709, #3 TRCN0000047710, #4 TRCN0000047711, #5 TRCN0000047712); VAV2 (#1 TRCN0000048223, #2 TRCN0000048224, #3 TRCN0000048225, #4 TRCN0000048226, #5 TRCN0000048227), and GEF-H1 (#1 TRCN0000003173, #2 TRCN0000003174, #3 TRCN0000003175, #4 TRCN0000003176, #5 TRCN0000010764) were from Open Biosystems (Huntsville, AL). Lentiviruses were prepared at the Lenti-shRNA Core Facility (UNC-Chapel Hill, NC).

### Western blot analysis

Western blotting was performed as described^20^. The use of gels/blots in figures complied the digital image and integrity standards as stated in the Scientific Reports editorial and publishing policies.

### In vitro Matrigel invasion assay

The PET membranes (8 µm pore size) of FluoroBlok™ cell culture inserts (351152, Corning, NY, USA) were coated with 90 µL of diluted Matrigel (0.3 mg/mL) (356234, Corning), and incubated at 37 °C for 2–3 h until solidified. Next, 1 × 10^4^ of 4T1, BT20 or MDA-MB231 cells suspended in serum-free DMEM media were seeded on the solidified Matrigel layer. Then, 700 µL of chemo-attractant medium (DMEM, 1% P/S and 10% FBS) was added to the lower chambers (353504, multiwell 24 well companion plate, Corning), and the plate was incubated in a 37 °C incubator. After 24 h of incubation, the insert bottoms were dipped in 1× PBS and stained in diluted Calcein AM (354217, Corning) in PBS for 10 min. Fluorescence images of invaded cells were captured with an EVOS inverted microscope, and analysis was done with ImageJ software.

### Immunofluorescence

Cells were plated on laminin-coated (Sigma, MO, USA) glass coverslips and grown until the desired confluency. Next, they were fixed with 100% methanol for 10 min, and incubated with the indicated primary antibodies followed by Alexa Fluor^®^ 546 secondary antibody (1:5000, ThermoFisher, MA, USA) and DRAQ5^®^ (1:2000, Cell Signaling Technology, MA, USA) nuclear staining. Fluorescence images were captured with a Leica TCS SP5 multiphoton laser scanning confocal microscope and E-cad intensity on cell–cell membranes was analyzed with MetaMorph software (Molecular Devices).

### Immunohistochemistry (IHC)

IHC staining of FFPE sections were performed as described^20^. Stained whole slides were scanned with an Olympus slide scanner and analyzed with ImageJ software.

### Mammary fat pad injection for spontaneous metastasis assay

The detailed procedure for mammary fat pad injection was as previously reported^20^. The Department of Laboratory Animal Resources at the University of Toledo Health Science Campus is accredited by the Association for the Assessment and Accreditation of Laboratory Animal Care International (AAALAC) and operates in full compliance with the OLAW/PHS policy on the Humane Care and Use of Laboratory Animals and the USDA Animal Welfare Act. All animal protocols used in this study were approved by the University of Toledo Institutional Animal Care and Use Committee (IACUC) and all experiments were performed in compliance with the ARRIVE guidelines and regulations in the approved protocols.

### RhoA and RhoGEF activities pulldown assay

The detailed protocol has been published^42^. Briefly, the pull-down tubes were set up to equalize both the total protein and total volume (1 mg/1 mL) and the lysate and the buffer was added into tubes with RBD/PBD beads (30 μg) or RhoAG17A beads (30 μg) for RhoA or RhoGEF actvitiy pulldown, respectively.

### Statistical analysis

Statistical calculations with two-tailed Student’s t-test were done using GraphPad Prism software. All the data are presented as means with error bars representing standard error.

## Supplementary Information

Supplementary Figures.

## Data Availability

No datasets were generated or analyzed during the current study.
