# Author Correction: The RhoA dependent anti-metastatic function of RKIP in breast cancer

**DOI:** 10.1038/s41598-023-38498-8

**Published:** 2023-07-12

**Authors:** Gardiyawasam Kalpana, Christopher Figy, Jingwei Feng, Claire Tipton, Julius N. De Castro, Vu N. Bach, Clariza Borile, Alexandria LaSalla, Hussain N. Odeh, Miranda Yeung, Rafael Garcia‑Mata, Kam C. Yeung

**Affiliations:** 1 Department of Cancer Biology, College of Medicine and Life Sciences, University of Toledo, Health Science Campus, Toledo, OH 43614 USA; 2 Department of Biological Sciences, College of Natural Sciences, University of Toledo, Toledo, OH 43614 USA; 3 Present Address: Department of Plastic and Cosmetic Surgery, Nanfang Hospital, Southern Medical University, 1838 Guangzhou North Road, Guangzhou, 510515 Guangdong People’s Republic of China

Correction to: *Scientific reports* 10.1038/s41598-021-96709-6, published online 31 August 2021

The original version of this Article contained an error in the legend of Figure 6b, where the citation of Ref. 20, as well as a disclosure for previous use of data, were omitted. The data shown in Fig. 6b was used previously in another article (Fig. 2d, *Scientific Reports* (2019) 9:16351 | 10.1038/s41598-019-52746-w) without disclosure. The duplicated data was used to confirm the RhoA expression was successfully knocked down in a breast cancer cell line that was used in both publications.

As a result,

“Increased RhoA expression is required for RKIP-mediated suppression of breast cancer metastasis in a murine allograft model. (**a**) (Left panel) Representative western blots of lysates prepared from control knockdown (shLUC), or RKIP knockdown (shRKIP^369^) 4T1 cells. (Middle panel) Weight of primary tumors harvested from Balb/c mice 30 days after orthotopical implantation with the indicated 4T1 cells shown in the left panel. (Right panel) Number of lung metastatic nodules recorded in Balb/c mice 30 days after orthotopical implantation with the indicated 4T1 cells shown in the left panel. n = 6. (**b**) Representative western blots of lysates prepared from control knockdown (shCTR), or three different RhoA knockdowns (shrhoA#32-34) 4T1 gfp-luc cells. (**c**) (Left panel) Representative ex vivo color images of lungs harvested from mice orthotopically injected with the indicated 4T1 gfp-luc cells showed in (**b**), (Right panel) Quantification of lung metastatic nodules (mean ± SE) detected in lungs harvested from mice orthotopically injected with the indicated 4T1 gfp-luc cells shown in (**b**). n = 4. (**d**) (Top panel) Representative ex vivo BLI images of lungs harvested from mice orthotopically injected with the indicated 4T1 gfp-luc cells shown in (**b**), (Bottom panel) Photon flux quantification of (mean ± SE) BLI images shown in the top panel. n = 4. (**e**) (Left) Representative hematoxylin and eosin (H&E) stained cross-sections of lungs harvested from mice orthotopically injected with the indicated 4T1 gfp-luc cells showed in (**b**) showing metastases demarcated from normal tissues with black solid line. (Right panel) total tumor metastases area calculated from the H&E sections shown above. n = 4. Unpaired Student's t-test (two-tailed) was used for all analyses with *p* < 0.05 considered significant. *ns* not significant.”

now reads:

“Increased RhoA expression is required for RKIP-mediated suppression of breast cancer metastasis in a murine allograft model. (**a**) (Left panel) Representative western blots of lysates prepared from control knockdown (shLUC), or RKIP knockdown (shRKIP^369^) 4T1 cells. (Middle panel) Weight of primary tumors harvested from Balb/c mice 30 days after orthotopical implantation with the indicated 4T1 cells shown in the left panel. (Right panel) Number of lung metastatic nodules recorded in Balb/c mice 30 days after orthotopical implantation with the indicated 4T1 cells shown in the left panel. n = 6. (**b**) Representative western blots of lysates prepared from control knockdown (shCTR), or three different RhoA knockdowns (shrhoA#32-34) 4T1 gfp-luc cells. The data shown in Fig. 6b was used previously in another article (Fig 2d)^20^. The duplicated data was used to confirm the RhoA expression was successfully knocked down in a breast cancer cell line that was used in both publications. (**c**) (Left panel) Representative ex vivo color images of lungs harvested from mice orthotopically injected with the indicated 4T1 gfp-luc cells showed in (**b**), (Right panel) Quantification of lung metastatic nodules (mean ± SE) detected in lungs harvested from mice orthotopically injected with the indicated 4T1 gfp-luc cells shown in (**b**). n = 4. (**d**) (Top panel) Representative ex vivo BLI images of lungs harvested from mice orthotopically injected with the indicated 4T1 gfp-luc cells shown in (**b**), (Bottom panel) Photon flux quantification of (mean ± SE) BLI images shown in the top panel. n = 4. (**e**) (Left) Representative hematoxylin and eosin (H&E) stained cross-sections of lungs harvested from mice orthotopically injected with the indicated 4T1 gfp-luc cells showed in (**b**) showing metastases demarcated from normal tissues with black solid line. (Right panel) total tumor metastases area calculated from the H&E sections shown above. n = 4. Unpaired Student's t-test (two-tailed) was used for all analyses with *p* < 0.05 considered significant. *ns* not significant.”

The original Article has been corrected.

