# Supplementary material for: The RhoA dependent anti-metastatic function of RKIP in breast cancer
Source: Sci Rep. 2021 Aug 31;11:17455. doi: 10.1038/s41598-021-96709-6 (PMC8408146; doi:10.1038/s41598-021-96709-6)
Supplement: Supplementary file 1 — Supplementary Figures. [file 41598_2021_96709_MOESM1_ESM.pdf]

Supp. Figure 1

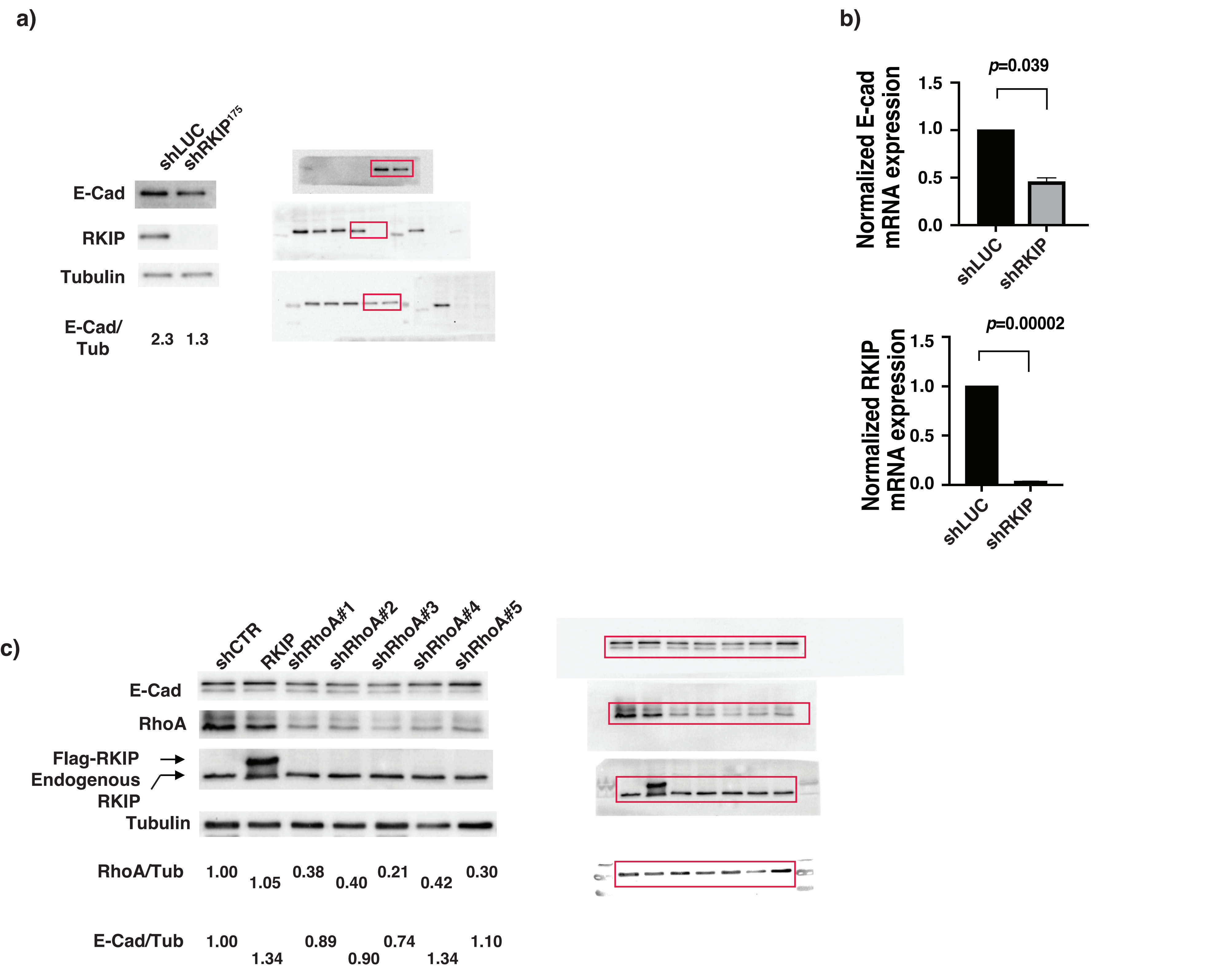

Supp. Figure 2

a)

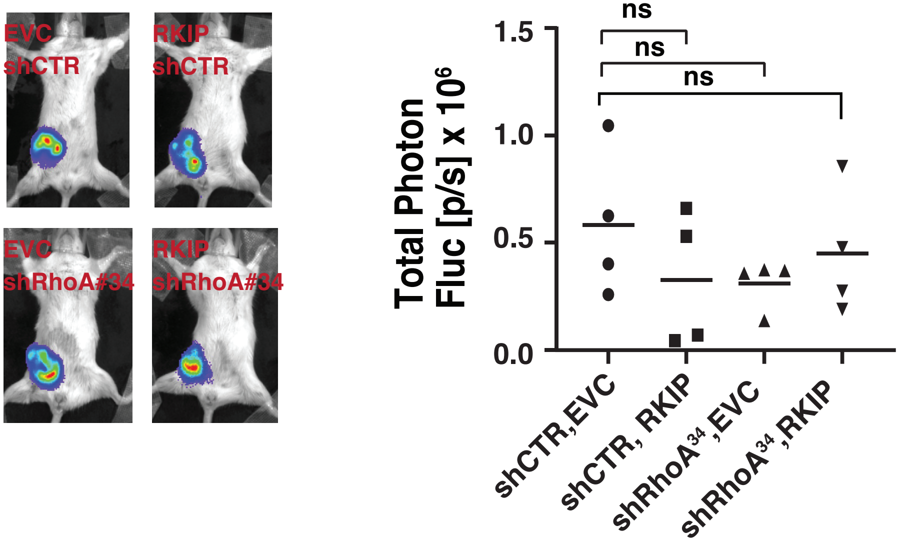

b)

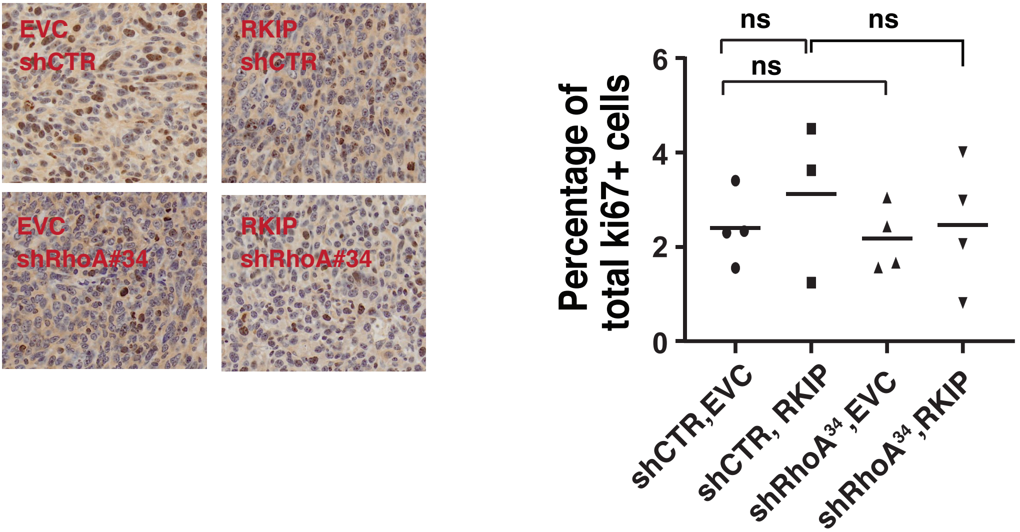

Supple Figure 3

a)

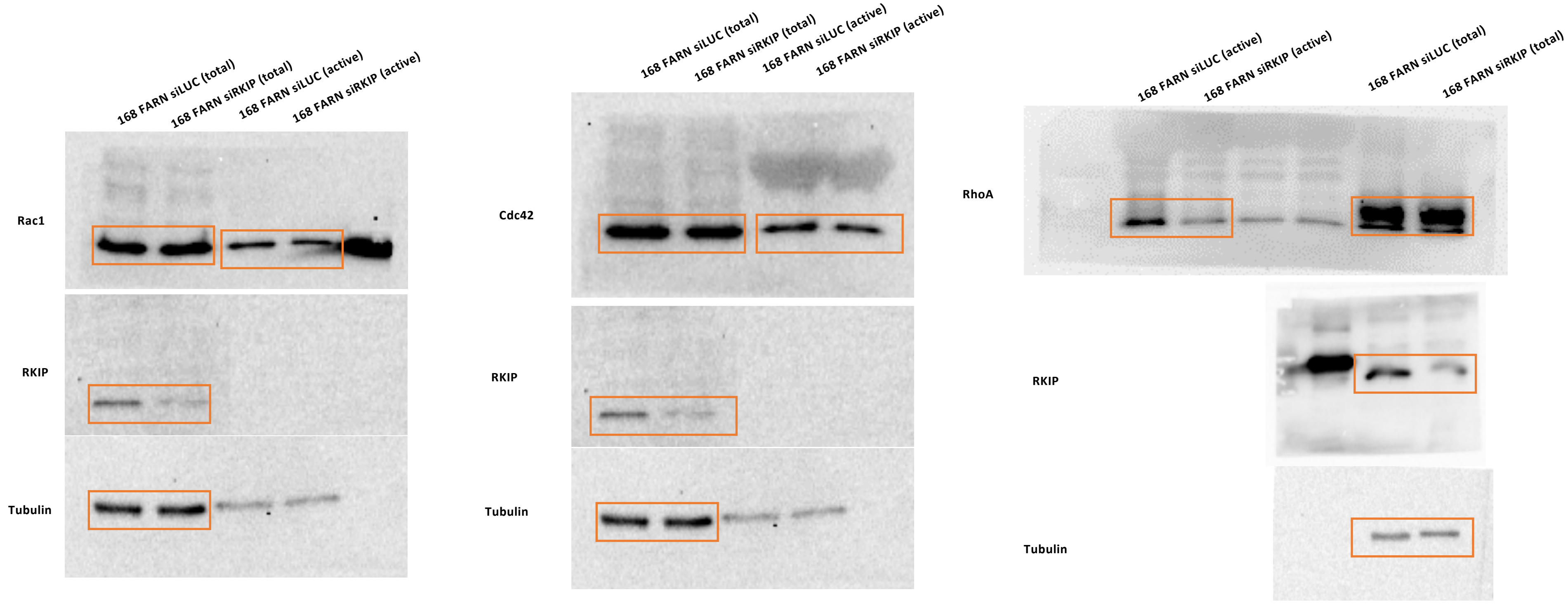

Supple Figure 3

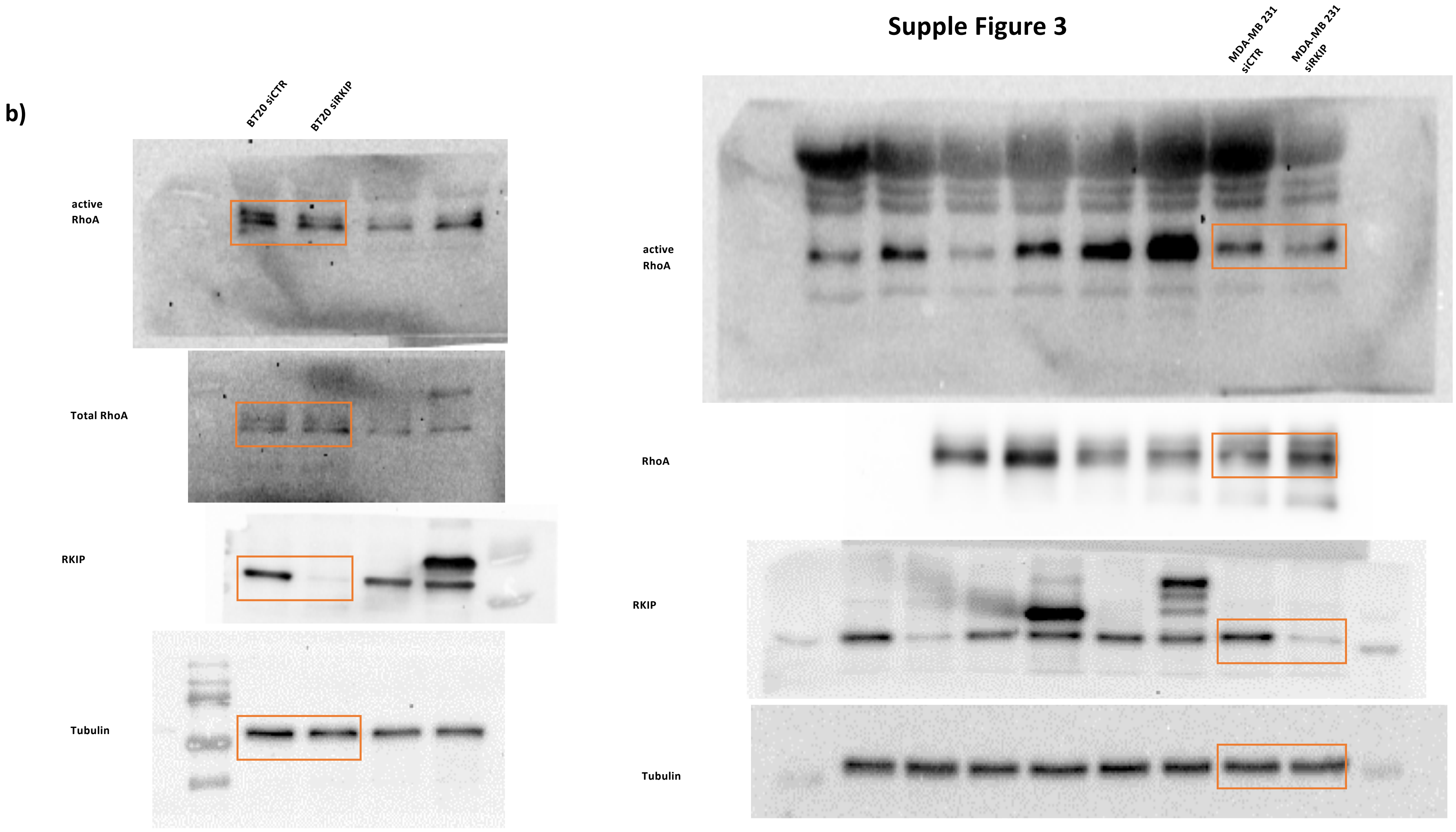

Supple Figure 3

c)

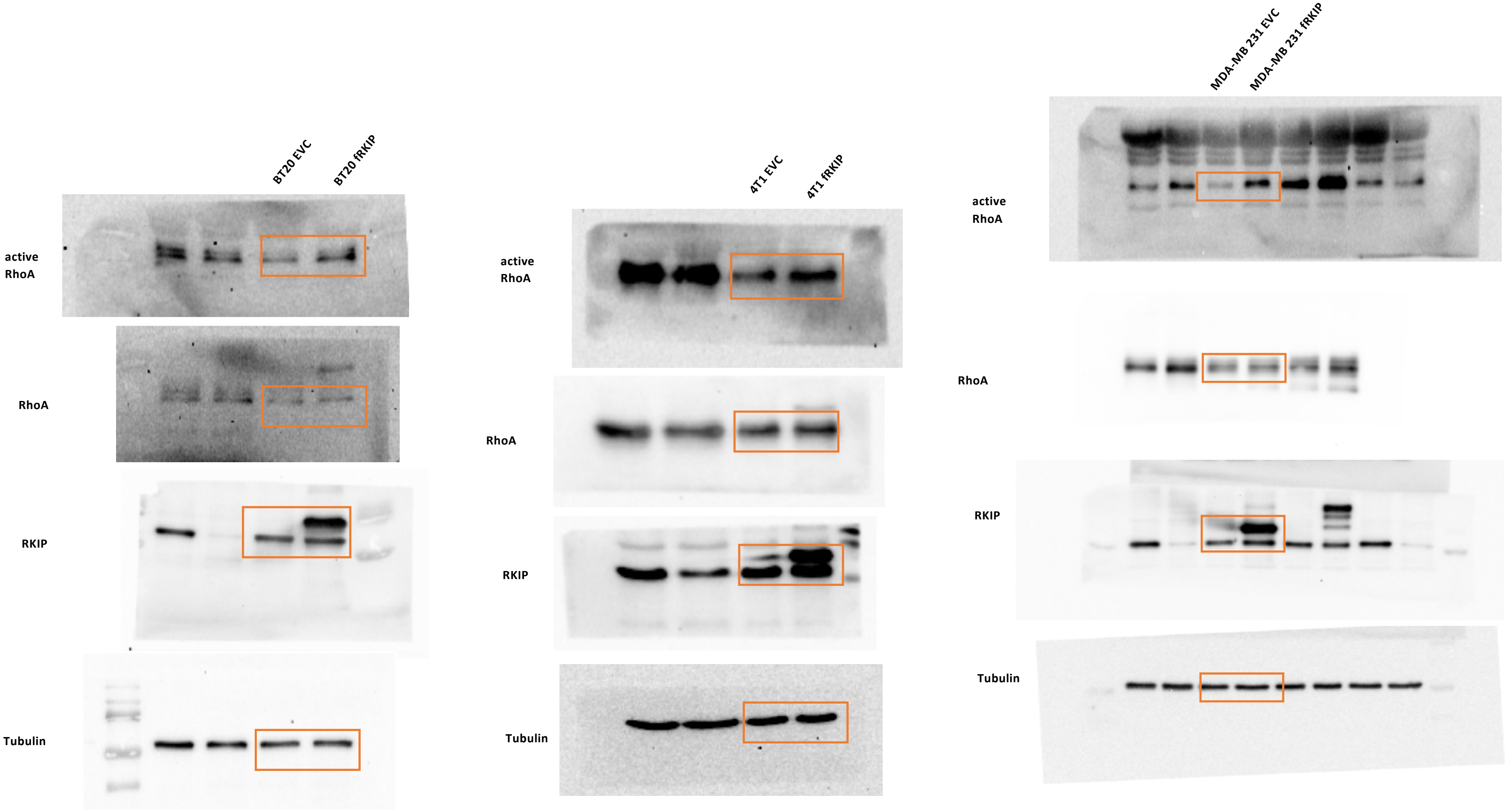

Supple Figure 3

d)

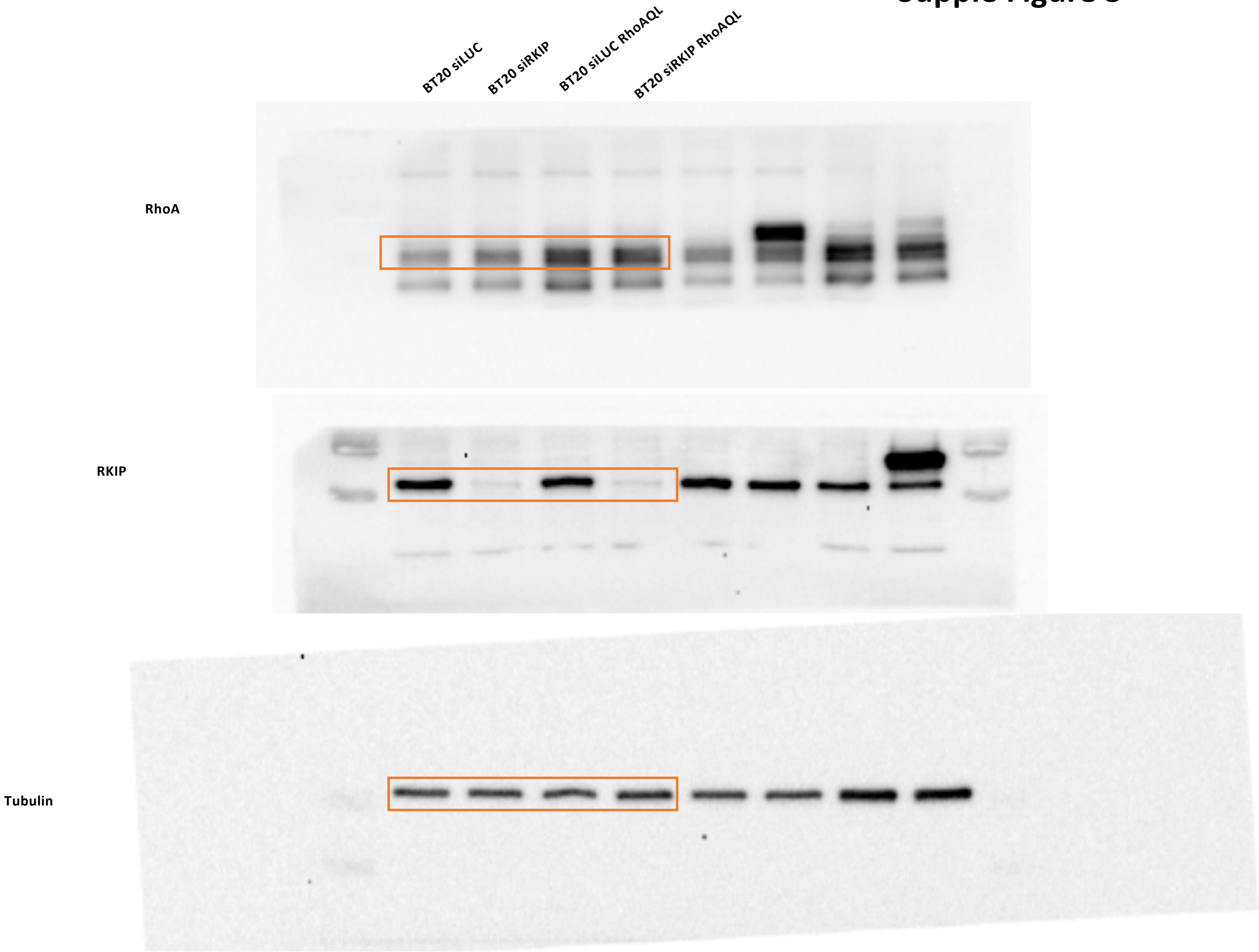

Supple Figure 4

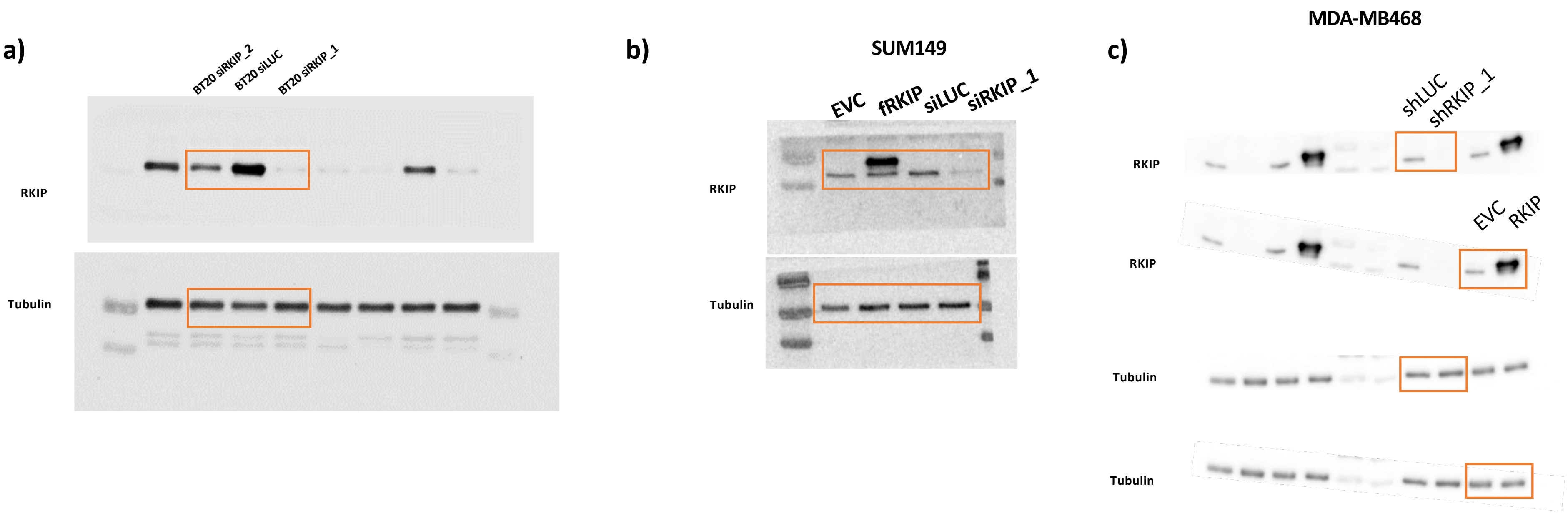

Supple Figure 4

d)

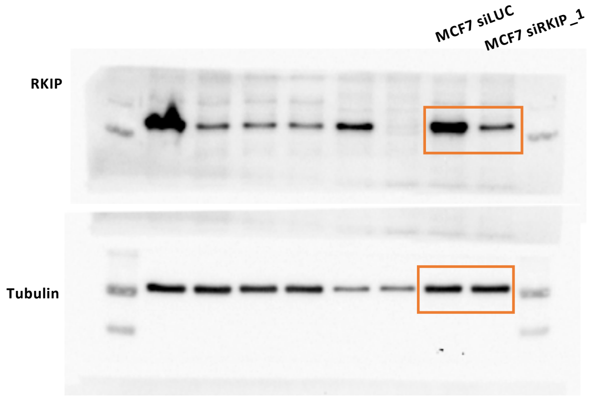

e)

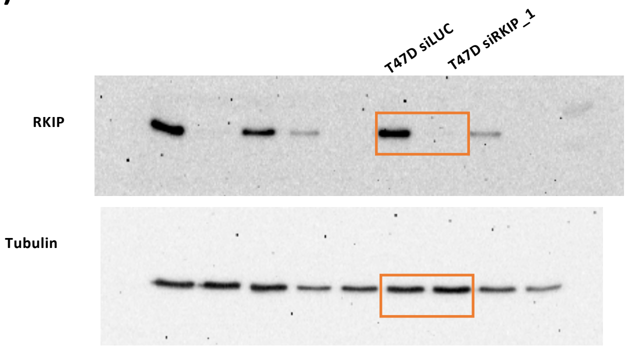

Supple Figure 4

f)

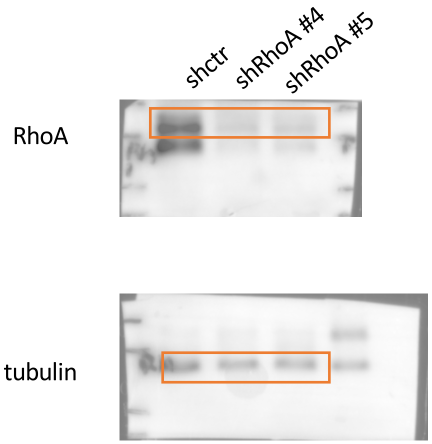

g)

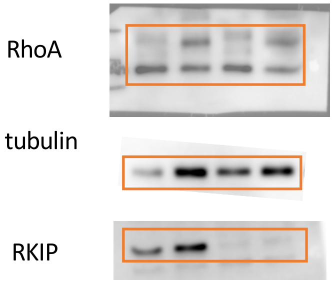

Supple Figure 5

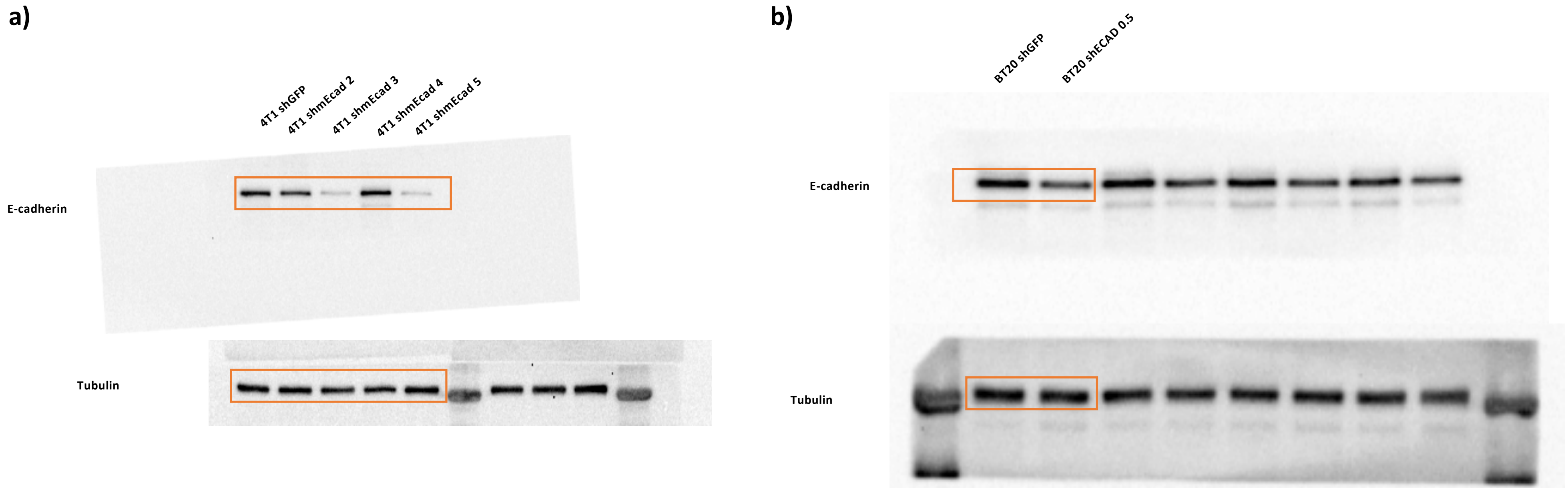

Supple Figure 5

c)

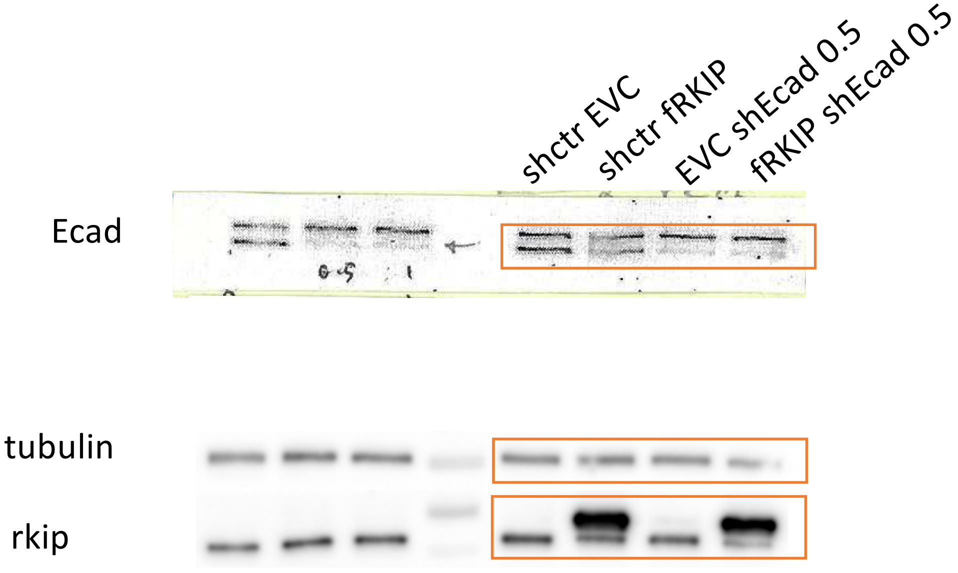

Supple Figure 6

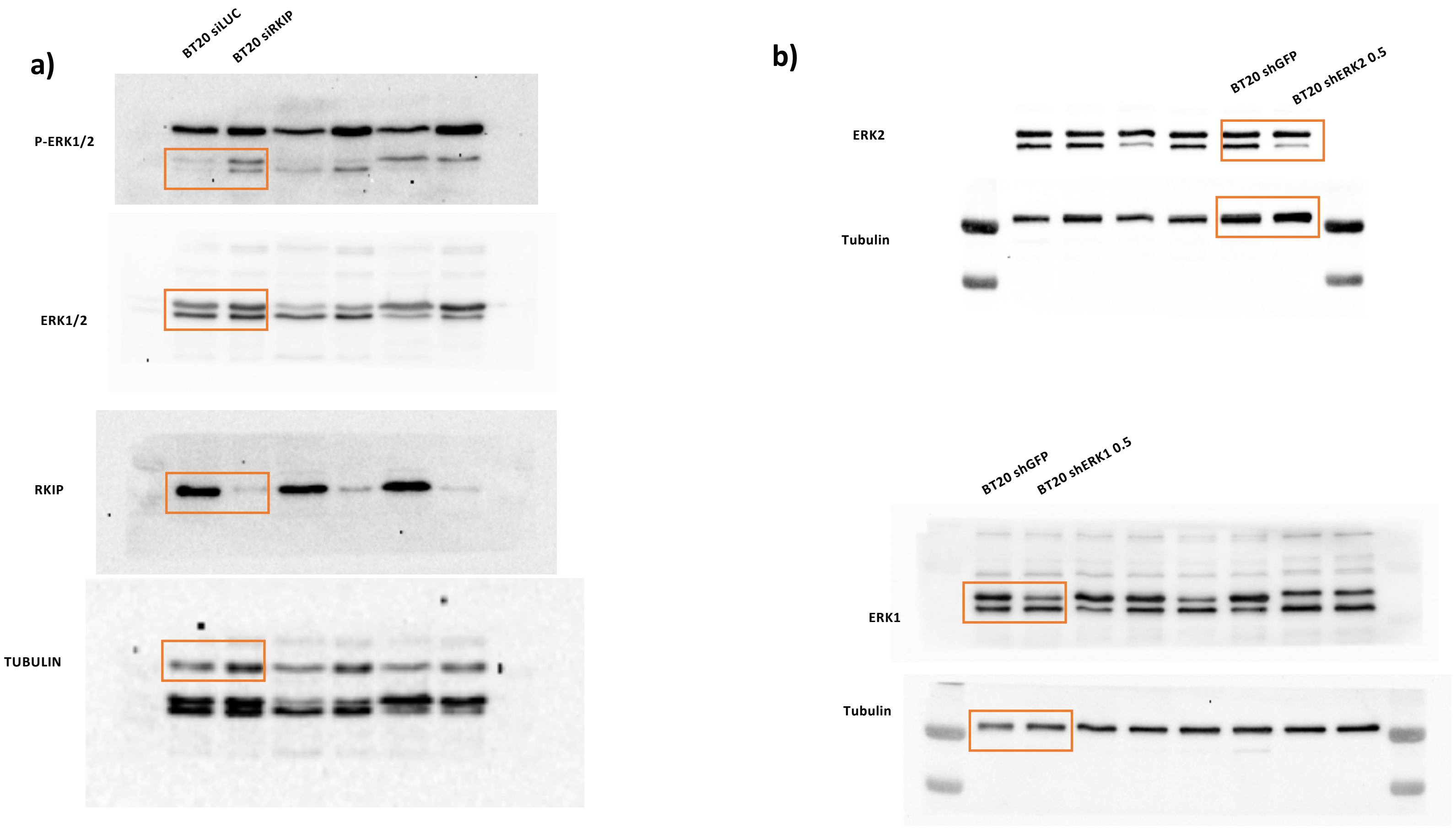

Supple Figure 7

a)

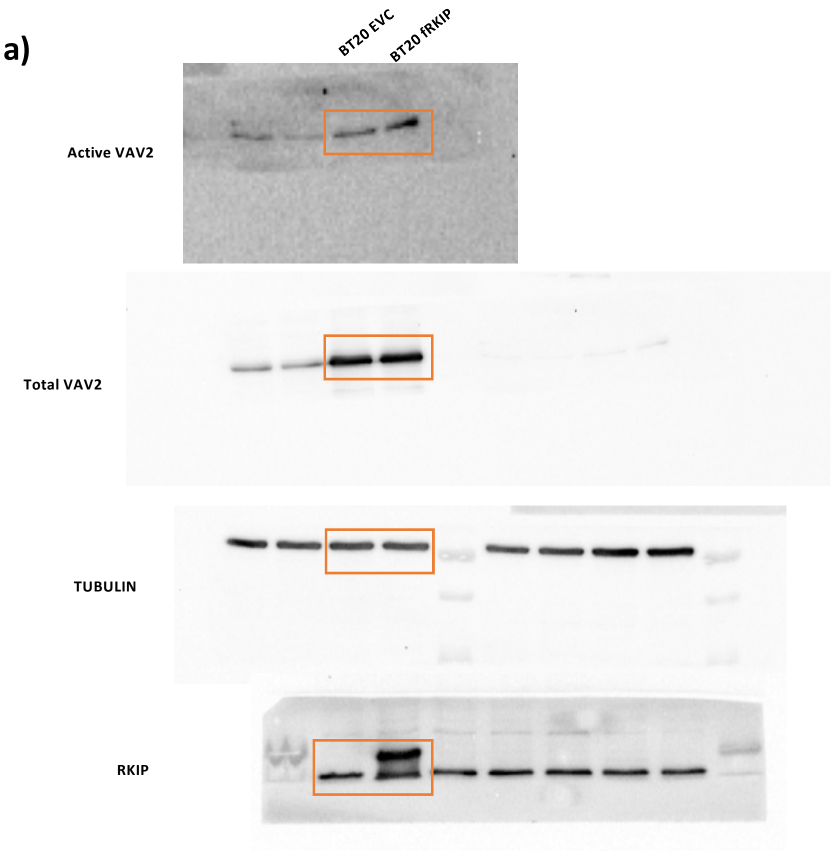

b)

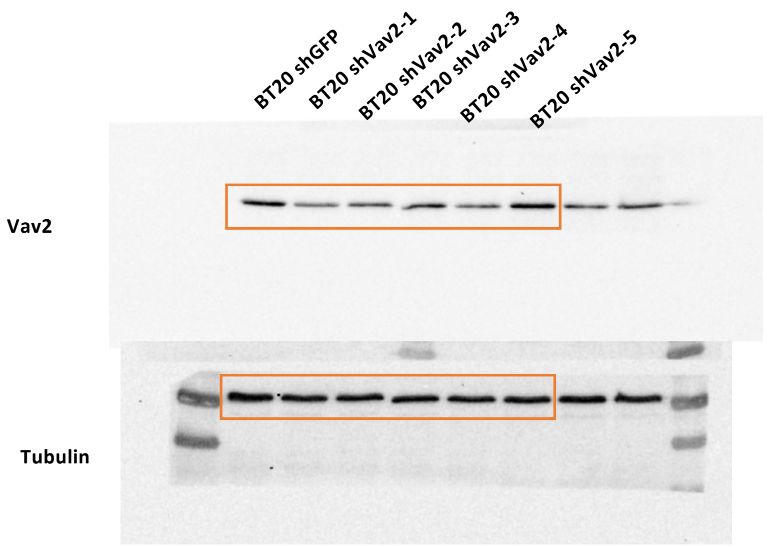

Supple Figure 7

c)

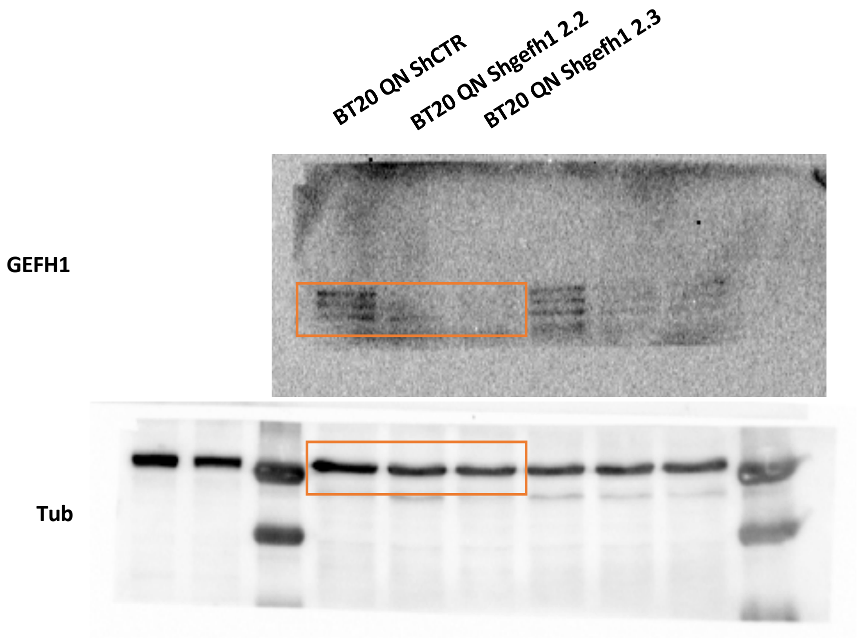

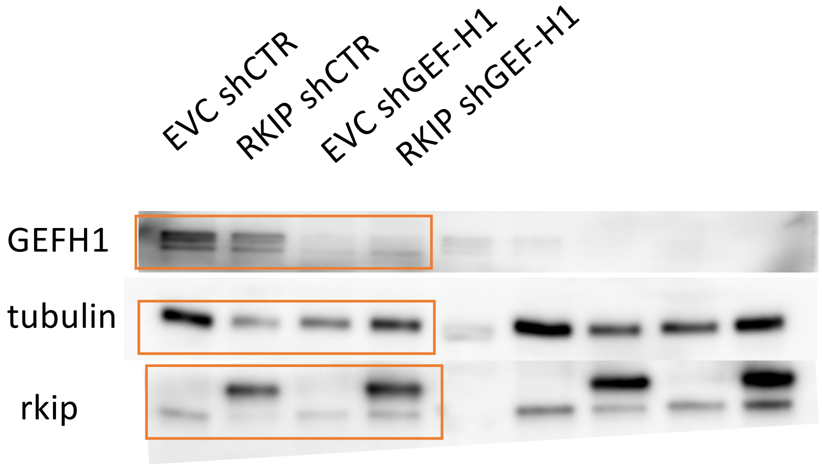

Supple Figure 8

a)

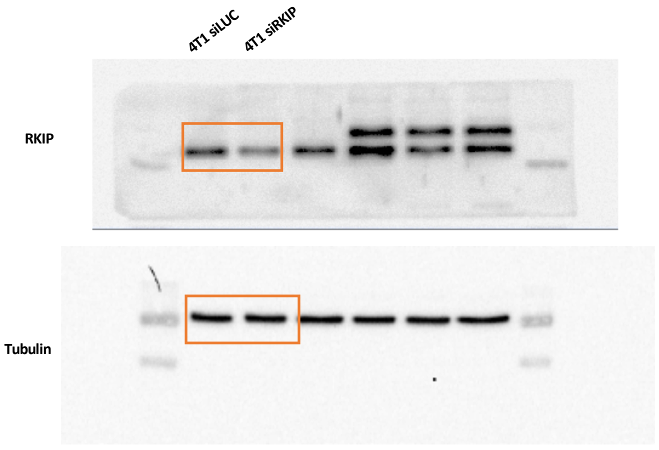

b)

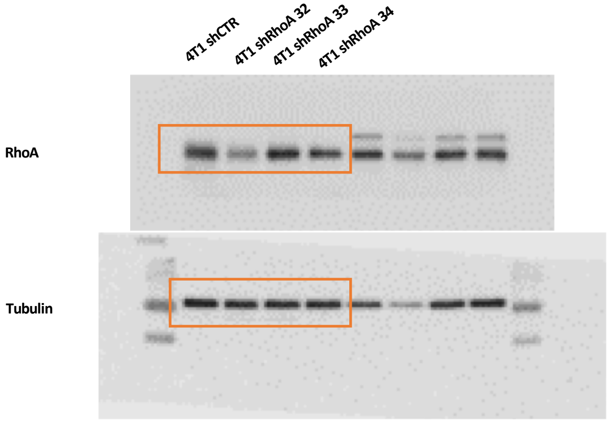

Supplementary Information

Figure Legends

Supplementary Figure 1. (a) Representative western blots of lysates prepared from control knock-down (shLUC) or RKIP knockdown ($shRKIP^{175}$) BT20 cells with the indicated Abs. Numbers are shown for quantified E-cad bands normalized with tubulin. (b) Relative E-cad (upper) or RKIP mRNA levels normalized with Actin (mean ± SE), as quantified by qRT-PCR in (shLUC) or RKIP knockdown ($shRKIP^{175}$) BT20 cells as indicated. Unpaired Student's t-test (two-tailed) was used for analyses with p<0.05 considered significant. (c) Representative western blots of lysates prepared from control knockdown (shCTR), RKIP expressing, or five different RhoA knockdowns (shrhoA#1-5) BT20 cells with indicated Abs. Numbers are shown for quantified RhoA or E-cad bands normalized with tubulin.

Supplementary Figure 2. (a) (left panel). Representative BLI images of mice 30 days after orthotopically injected with the indicated 4T1 gfp-luc cells. (right panel) Photon flux quantification of (mean ± SE) BLI images shown in panel a. n=4. (b) (left panel). Representative immunohistochemical (IHC) E-cad Ab staining images of breast primary tumor sections of mice orthotopically injected the indicated 4T1 gfp-luc cells. (right panel). Quantification of areas per tumor field of view (FOV) stained positive in the right panel for ki-67. n=4. Unpaired Student's t-test (two-tailed) was used for all analyses with p<0.05 considered significant. ns: not significant.

Supplementary Figure 3. The complete blots from the western analysis performed in Figure 1. The red rectangle is showing the cropped region of the blots included in the figure.

Supplementary Figure 4. The complete blots from the western analysis performed in Figure 2. The red rectangle is showing the cropped region of the blots included in the figure.

Supplementary Figure 5. The complete blots from the western analysis performed in Figure 3. The red rectangle is showing the cropped region of the blots included in the figure.

Supplementary Figure 6. The complete blots from the western analysis performed in Figure 4. The red rectangle is showing the cropped region of the blots included in the figure.

Supplementary Figure 7. The complete blots from the western analysis performed in Figure 5. The red rectangle is showing the cropped region of the blots included in the figure.

Supplementary Figure 8. The complete blots from the western analysis performed in Figure 6. The red rectangle is showing the cropped region of the blots included in the figure.
